# A multimodal microfluidic-based platform integrating topographical and equibiaxial mechanical cues for next-generation *in vitro* cell microenvironment mimicking

**DOI:** 10.3389/fbioe.2025.1657107

**Published:** 2025-10-17

**Authors:** Denise Pagliara, Raffaele Vecchione, Valentina Mollo, Paolo Antonio Netti

**Affiliations:** ^1^ Department of Chemical, Materials and Industrial Production Engineering (DICMAPI), University of Naples “Federico II”, Naples, Italy; ^2^ Center for Advanced Biomaterials for Health Care (CABHC), Italian Institute of Technology (IIT), Naples, Italy; ^3^ Interdisciplinary Research Centre on Biomaterials (CRIB), University of Naples “Federico II”, Naples, Italy

**Keywords:** microenvironment, lab-on-a-chip, microfluidics, biomechanics, cardiomyocytes

## Abstract

The cellular microenvironment is a powerful regulator of the cell state and function. Both biochemical and morphophysical environmental cues have been shown to profoundly influence cellular decisions. However, the fundamental principles governing the intricate crosstalk between microenvironmental manipulation and the modulation of cell functions remain largely elusive. To unravel the regulatory role of the microenvironment in determining cellular fate and state, it is essential to develop tools capable of precisely presenting and integrating these signals. In this context, we propose a next-generation cell culture system that synergistically combines microfluidic and biomechanical platforms. This system is designed to systematically deliver microenvironmental stimuli to condition cell state. As a notable use case, we selected cardiomyocytes (CMs) given the well-documented influence of biochemical and morphophysical cues on cardiac tissue homeostasis. The platform features a multilayer design integrating complex mechanical stimulation, such as equibiaxial strain, on a deformable membrane equipped with microchannels for nutrient delivery. A radial micropattern was fabricated on the membrane to guide cell alignment along the direction of stretching, thereby homogenizing cellular response. The functionality of the device was first validated through COMSOL simulations and subsequently experimentally tested to confirm the interplay between equibiaxial mechanical stimulation and fluid flow. When HL-1 rat atrial CMs were seeded on the platform, they proliferated, aligned with the micropattern, and exhibited persistent migration along the stretching direction under equibiaxial deformation. These findings demonstrate that the combination of microenvironmental signals is critical for enhancing cellular activity and underscore the importance of accurately replicating the cell microenvironment in lab-on-chip applications.

## 1 Introduction

Research is currently focusing many efforts on recapitulating biological processes associated with human health at a laboratory scale with the final aim of preserving wellness and extending human lifespan. For this purpose, because cells are the fundamental morphological and functional units of the human body, they are extensively used as *in vitro* models to investigate biological processes. State-of-the-art studies have demonstrated that a deeper understanding of human health requires comprehending how the microenvironment influences cell behavior (Mazzio and Soliman, 2012). This investigation underscores the need to study how cells adapt to and interact with surroundings, as cells *in vivo* are embedded in a complex and dynamic interplay of biochemical and biophysical cues that collectively form the cell microenvironment. The microenvironment serves as a structural support for cell organization and assembly, heavily relying on the dynamic presentation and combination of biochemical and biophysical cues, including nano- and micro-topographies resulting from cell arrangement and the physical architecture of the extracellular matrix (ECM) (Riching et al., 2015; Huang et al., 2017). In addition to biochemical and topographical stimulation, cells detect and react to mechanical cues depending on the type of tissue, for example, cyclical tensile stress in heart and lungs, and mainly compressive stress in cartilage and bone (Waters et al., 2002; Sommer et al., 2015; Chelnokova et al., 2016; Lawless et al., 2017; Eschweiler et al., 2021; Marchioni et al., 2021). This highlights the need to employ deformation units to reproduce the *in vitro* mechanical identity of cells. In addition, cells *in vivo* are very precisely organized and oriented in the tissue architecture, whereas standard cultures result in randomly arranged cells. Cells stimulated mechanically in different directions exhibit distinct responses, as the biosynthetic response of cells is governed by their orientation relative to the direction of deformation and the consequent activation of distinct mechanotransduction pathways (Kumar et al., 2002; Wang et al., 2004; Chagnon-Lessard et al., 2017). Consequently, a random arrangement of cells on the seeding substrate results in significant heterogeneity in the response of the stimulated cell population, due to a delayed response between shape and traction forces reorientation (Krishnan et al., 2012). Although cells aligned with the direction of uniaxial mechanical deformation exhibited optimal performance in terms of cell organization and functionality (Wang et al., 2014; Jafari et al., 2023; Kim et al., 2023), research conducted on various cell lines demonstrated that the uniaxial deformation is typically affected by the Poisson effect. Therefore, a stretch along the principal axis is accompanied by a contraction perpendicular to the direction of strain, which can influence the downstream cellular response, resulting in increased heterogeneity in the cell population (Zhao et al., 2011; Shao et al., 2014; Tamiello et al., 2016; Melo-Fonseca et al., 2023). For this reason, equibiaxial stretching has been implemented in diverse cell culture applications to achieve a more homogeneous response (Butcher et al., 2006; Moraes et al., 2009; Gould et al., 2012), although it has not yet been integrated into a microfluidic system. It has also been shown that this stimulation, when applied alone with randomly distributed cells, can lead to cellular disorganization and impaired tissue functionality (Van Spreeuwel et al., 2014). Therefore, it is necessary to combine complex equibiaxial stretching with the proper spatial arrangement of the cells, both to ensure a homogenous cellular response to the mechanical stimulation and, on a general perspective, to accurately reproduce the biophysical identity of the tissue of interest (Thompson et al., 2020). To date, no studies have reported the combination of equibiaxial mechanical stimuli and homogeneous geometrical confinement of cells, nor on the application of these biophysical stimulations in a miniaturized context, in which a microfluidic-integrated system interfaces these biophysical cues with the biochemical ones. This is crucial, as a miniaturized, engineered system capable of combining and controlling these cues would leverage the inherent advantages of microfluidic technology, such as precise control of experimental conditions, rapid adjustment of parameters, real-time monitoring, minimal culture media consumption, and parallelization capability (Sackmann et al., 2014; Leung et al., 2022). These features collectively enhance the biological fidelity of the *in vitro* model for microenvironment reproduction, paving the way to technological advancements in lab-on-chip applications.

Due to the complexity of its microenvironment, which includes mechanical loading and high structural organization, cardiac on-chip applications represent a relevant case study where the primary aim is to ensure cardiomyocytes (CMs) proliferation, survival and coordinated activity (Parker and Ingber, 2007; Sun et al., 2019; Wu et al., 2020; Liao et al., 2021; Ingber, 2022). A comprehensive knowledge of the individual and combined effects of different biophysical microenvironmental signals on the cardiac cell population remains limited to a few studies. For instance, Navaee et al. demonstrated that coupling uniaxial mechanical stimulation with anisotropic topographies induced off-axis alignment of cardiac cells, mimicking the heart’s helicoidal architecture (Navaee et al., 2023). However, uniaxial mechanical cues are insufficient to mimic the complex myocardial tissue deformation field. Siddique et al. employed a circular diaphragm to integrate both uniaxial and equibiaxial strains with uniaxial alignment, revealing that equibiaxial stimulation best supports CMs growth and structural organization (Siddique et al., 2022). Despite the promising outcomes of this work, it relies on uniaxial cell alignment that do not fully capture the heart's complex functionality, which instead is characterized by competing local myocardial anisotropy and global helicoidal tissue organization (Bijnens et al., 2012; Dwyer and Coulombe, 2021; Navaee et al., 2023). Additionally, current macroscopic platforms lack the advantages offered by miniaturized systems, which are valuable solutions in the integration of multiple microenvironmental factors, including mechanical stimulation, topographical cues and fluid flow, essential for physiologically relevant *in vitro* models.

In this work, we propose a novel microfluidic approach to implement a set of microenvironmental signals on a single platform, to assess cell health and function in an *in vitro* context. In particular, the combined effect of mechanical deformation field generation and structural topography was analyzed in the platform, focusing on replicating the cardiac microenvironment. This microfluidic-integrated approach potentially extends beyond simple cell cultivation on a chip by applying the biophysical stimuli alongside biochemical fluxes, simplified in this study to nutrient flow for cell feeding and survival. A multilayered platform enabled the integration of equibiaxial mechanical stimulation on a deformable membrane, along with fluid flow microchannels. Photolithography was employed to fabricate a radially arranged micropattern on the membrane surface, aligning cells along the direction of stretching, thereby providing uniform cell stimulation and clarifying the role of the combined equibiaxial and topographical stimulation on cell behavior. The device’s functionality was first verified through COMSOL simulations and then experimentally tested. HL-1 rat atrial CMs were seeded on the platform due to their ability to exhibit gene expression profiles similar to adult CMs, while maintaining a contractile cardiac phenotype and functionality (Claycomb et al., 1998). The results demonstrated that the proposed integrated multi-signals platform, in action, successfully promoted HL-1 cells survival and proliferation. HL-1 cells aligned according to the substrate radial micropattern and homogenously migrated along its direction when topography was combined with continuous equibiaxial deformation, indicating the importance of complementing different biophysical cues in the cell stimulation. This study underscored the high sensitivity of cell behavior to the combination of multiple signals, as well as the potential of the proposed platform to effectively present and control different stimuli representative of the cell microenvironment.

## 2 Results

### 2.1 Design and fabrication of the microfluidic platform

**FIGURE 1:**
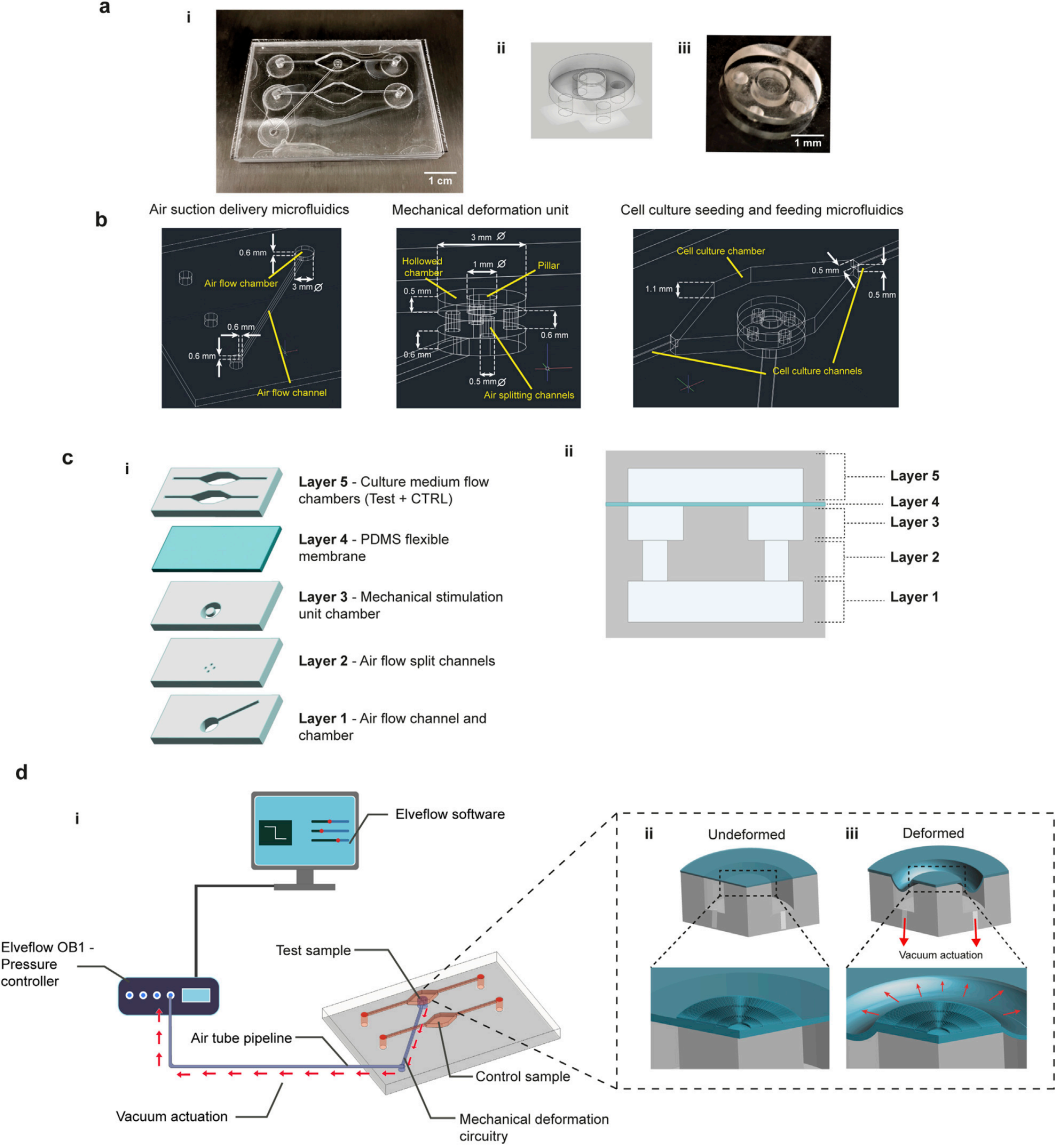
Microfluidic platform design and functionality. A schematic representation of the platform reveals the separation of the air and fluid flow chambers and channel, test and control sample channels **(a-i)**, while a photograph of the platform helps to appreciate the transparency of the construct. A schematic representation and a photograph **(a-ii and a-iii)** of the mechanical deformation unit is also highlighted. **(b)** The characteristic dimensions of the device channels and chambers are reported, specifying the dimensioning of the three main sections, i.e., air suction delivery, mechanical deformation unit and cell culture microfluidics. **(c)** The platform is characterized by a multi-layered stack of channels and chambers. From the bottom, the air suction unit leads the mechanical stimulation (layer 1,2 and 3), while a top layer is deputed to the fluid flow (layer 5). In the middle, a deformable PDMS membrane separates the air and fluid flow and undergoes the mechanical deformation, giving the substrate for cell seeding (layer 4). In **(c-ii)** a 2D section of the deformation chamber with the different layers is reported. **(d-i)** The platform is externally connected with a pressure controller (OB1 MK4, Elveflow) by a tubing system, remotely controlled by software. The deformable membrane is enriched with a surface micropatterning in correspondence to the deformation pillar **(d-ii)** and it undergoes an equibiaxial stretching when the membrane is pulled in the hollow chamber by the vacuum suction **(d-iii)**.

The device features a two-chamber design, allowing for simultaneous testing of stimulated and control cell cultures. The complete device is shown in Figure 1a. The platform structure consists of a lower part designed for the mechanical stimulation (blue channels in Figure 1a-i, inset in Figure 1a-ii and Figure 1a-iii), and an upper part for nutrient flow and waste removal in the cell culture (red channels, Figure 1a-i). A description of the individual layers and their roles in the platform is following (Figure 1c). The first layer consists of an air flow channel that delivers vacuum from the stimulation chamber to the outside, connected to an air chamber that distributes negative pressures for suction beneath the mechanical stimulation unit. The second layer includes four small channels for air splitting and enhancement of negative air pressures, symmetrically arranged at the bottom of the upper chamber. The third layer consists of a circular chamber with a central rigid pillar. The holes of the second layer align with both the underlying air vacuum chamber and the upper hollow circular chamber, ensuring symmetrical air suction, as represented in the lateral cross-section of the device (Figure 1c-ii). The deformable polydimethylsiloxane (PDMS) substrate is a thin membrane attached to the top of the deformation chamber. The membrane is in contact but not attached to the rigid pillar, allowing it to slide laterally without deflecting into the cavity under vacuum pressure. In addition, to facilitate the sliding of the membrane over the poly(methyl methacrylate) (PMMA) deformation pillar, its centre was hollowed out, leaving only a circular crown along the edge. This design reduced the contact area between the PDMS and the PMMA to this narrow layer, allowing the membrane to slide freely upon vacuum-induced stretching. The final layer of the platform houses flow channels and chambers for culture medium delivery to both test and control samples. The high aspect ratio design of the chambers minimizes turbulence, reducing bubble formation and shear stress on the cell culture in the deformation area, even at high flow rates (Supplementary Figure S1). In the test sample chamber, the PDMS membrane was enriched with a surface patterning, in the form of stripes arranged radially to match the circular shape of the underlying pillar (inset in Figures 1d-ii,iii).

Mechanical stimulation is achieved via air suction applied throughout the vacuum channels and chamber connected to the outside with a pressure controller and a software for pressure curves programming (Figure 1d-i, additional information in Section 5). The vacuum delivered through the four symmetric holes at the base of the hollow circular chamber pulls the lateral part of the membrane into the cavity, causing a vertical movement on the free side of the chamber, while the central pillar stops further lowering. As a result, the membrane undergoes controlled planar biaxial stretching as it slides over the pillar’s surface (Figure 1d-iii). The lateral stretch is equal along the two main planar axes, due to the symmetric arrangement of the four air flow split channels at the base of the deformation chamber. At the same time, the micropattern of the PDMS membrane undergoes simple stretching under the equibiaxial stimulation, without torsion or combined deformation field as it is oriented along the main stretching direction at each point among the circular pillar.

### 2.2 Simulative and experimental validation of the mechanical stimulation

**FIGURE 2:**
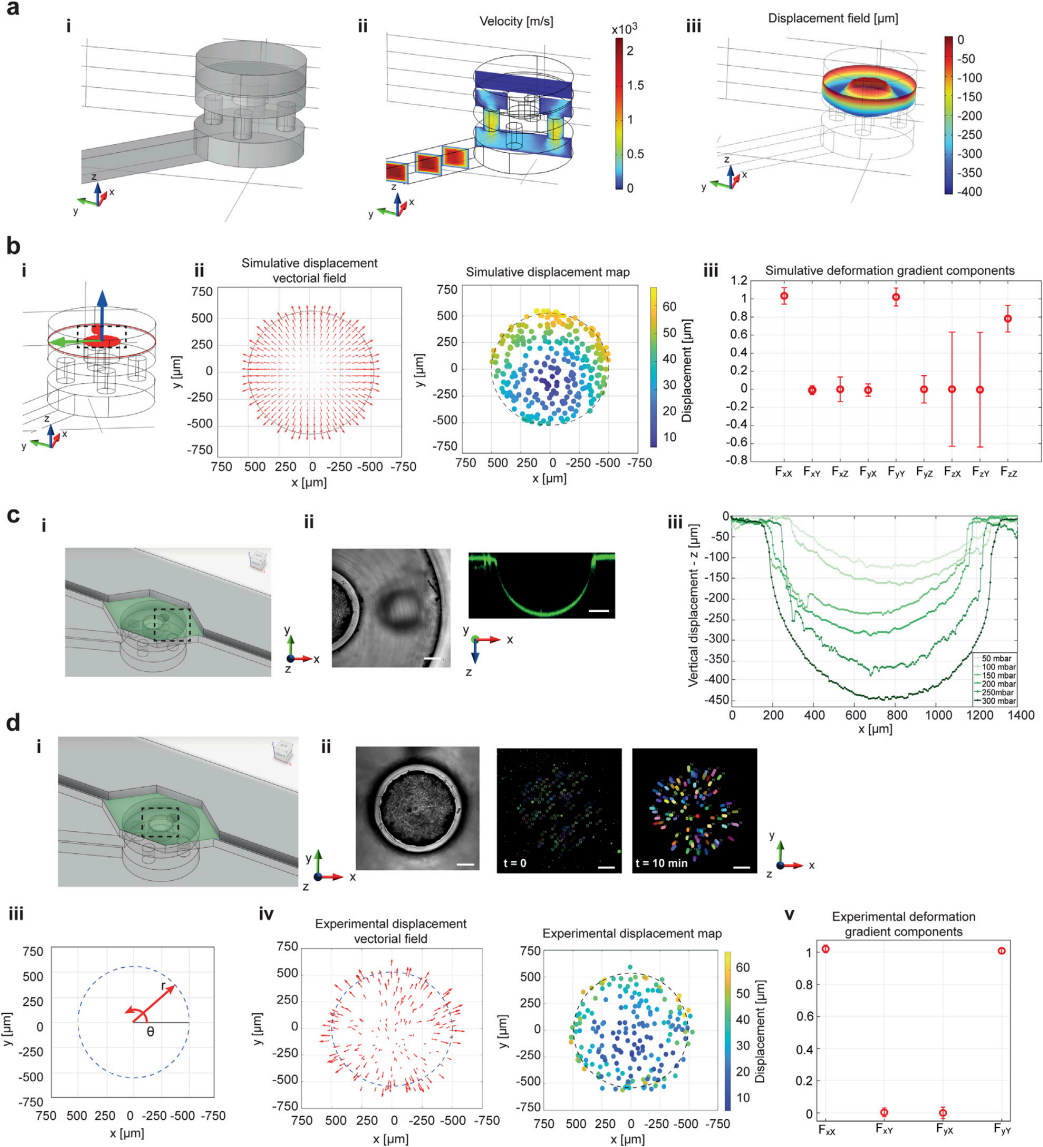
COMSOL simulations are compared to the experimental characterization of device working principle. The simulations were performed to prove the effect of the combination of microfluidic air flow and deformation mechanics on the PDMS membrane. An inset of the 3D deformation chamber area is reported in panel **(a-i)**. **(b)** 3D simulations of the air flow velocity proved the splitting ability of the small air flow channels, which increased the air velocity beneath the PDMS membrane **(a-ii)**. This corresponded to a vertical lowering of the PDMS flexible membrane in the hollow cavity **(a-iii)**. **(b)** The bending of the PDMS membrane over the fixed central pillar is inducing a deformation on the membrane surface in correspondence to the circular pillar, from which an equibiaxial deformation field can be recognized from vectorial field of the displacements (**b-ii**, left panel). A deformation gradient arises on the surface of the PDMS membrane, as demonstrated by the displacement map (**b-ii**, right panel). The deformation gradient has non-null elements only on the principal diagonal **(iii)**. Experimental tracking of membrane deformation was performed by embedding fluorescent nanobeads in the PDMS membrane. **(c)** A side view of the PDMS lowering, in correspondence to the hollow cavity of the deformation chamber **(i)**, is reported in brightfield (**ii**-left panel) and fluorescence images (**ii**-right panel). **(b-iii)** The vertical lowering of the flexible PDMS membrane was calibrated in the deformation chamber unit. **(d-i,ii)** Representative images of PDMS-embedded nanobeads position at time 0 (**ii**-central panel) and after 10 min (**ii**-right panel) on the pillar surface are reported. **(d-iii)** Polar coordinate system on the pillar surface helped for the description of the equibiaxial deformation field distribution. The PDMS membrane sliding over the pillar during equibiaxial stimulation was demonstrated by the displacement vectorial field (left panel **d-iv**) and the deformation map (right panel **d-iv**). A deformation gradient arose on the PDMS membrane when equibiaxial stimulation was applied **(d-v)**. The values of deformation gradient components are represented as mean ± S.D. Scale bar 200 μm.

Numerical methods were integrated into this study for a comprehensive analysis of the microfluidic platform functioning. In the 3D COMSOL simulations, the air flow channels and chambers were modeled to deliver vacuum to the PDMS membrane (Figure 2a-i; Supplementary Figure S2). The membrane was represented as a cylinder with a height of 40 μm and a diameter matching that of the deformation chamber, to replicate the actual dimensions of the testing device. As expected, the velocity profile indicated high speed suction in the small microfluidic channel and air-splitting holes (Figure 2a-ii; Supplementary Figure S3b). Due to the negative pressure difference between the inlet of the air flow channel and the base of the PDMS membrane (Supplementary Figure S3c), the solid mechanics simulation on the PDMS membrane revealed a vertical displacement field along the z-axis within the hollow cavity of the deformation unit (Figure 2a-iii). At the top of the pillar, the vertical displacement field was zero, as the pillar was treated as a fixed constraint for z-motion, though not for x-y displacement. The simulations demonstrated that the areas of maximum stress and strain were located at the boundaries, i.e., where the membrane bends at the fixed constraints, and along the sides of the pillar, where the PDMS membrane both bends and stretches (Supplementary Figures S4b,c). Focusing on the pillar surface, the principal strain map was also assessed to observe PDMS deformation in the area affected by the equibiaxial loading (black-dotted area in Figures 2b-i). Here, a gradient of deformations emerged from the center toward the pillar’s boundaries, represented by arrows whose orientation indicated the direction of deformation and the length represented its magnitude (Figure 2b-ii, left panel). In addition, the observed deformation was visibly equibiaxial and symmetric in all directions. Accordingly, the deformation magnitude was plotted as a displacement color map, demonstrating that a gradient of deformation arose on the pillar surface (Figure 2b-ii, right panel). The deformation gradient of the PDMS membrane under stretch showed non-null components only along the principal diagonal (components F_xX_, F_yY_ and F_zZ_ in Figure 2b-iii), meaning that rotations and shear forces did not contribute to the deformation (Supplementary Material, 2D plots in Supplementary Figure S5 and 1D line plots in Supplementary Figure S6). The only significant transformation of the membrane occurred through pure stretching.

To experimentally validate the platform’s functionality, the deformability of the flexible PDMS membrane was tested. Fluorescent nanobeads were embedded in the PDMS, and the OB1 external setup for pressure control was used to deform the fluorescently tagged PDMS membrane through air suction. A 40 μm-thick membrane was employed to ensure consistent deformability of the material (Supplementary Figure S10a). Downward displacement of the membrane into the hollow cavity of the deformation chamber was observed (Figures 2c-i,ii). As expected, the lowering of the PDMS membrane increased with higher air suction pressures (Figure 2c-iii); however, the relationship between vertical displacement and inlet pressure did not follow a linear trend (Supplementary Figure S10b-ii). To assess the deformation field on the pillar surface, the displacement of PDMS-embedded fluorescent nanobeads was tracked over time. The positions of the nanobeads were recorded every minute, starting at the onset of deformation, over a total observation period of 10 min (Figures 2d-i,ii). A deformation distribution was generated from the initial and final positions of the nanobeads’ trajectories on the pillar surface. Arrow plots showed that the direction of deformation followed the equibiaxial stimulation and was distributed along the θ-direction in polar coordinates centered on the circular pillar (Figures 2d-iii,iv, left panel). A displacement map, based on the nanobeads trajectories, demonstrated a variable displacement field, i.e., a deformation gradient, arising during the PDMS membrane deformation (Figure 2d-iv, right panel). Lower displacement values were observed at the center, increasing along the r-direction toward the pillar boundaries. These findings validated the simulation results of the PDMS membrane's mechanical deformation and the corresponding deformation gradient map in Figure 2b-ii. It was also evident that the PDMS membrane-embedded nanobeads experienced greater displacement on the pillar as the suction pressure increased (Supplementary Figure S10c). The quantification of the experimental deformation gradient components over the pillar surface validated the simulative results, confirming a pure stretching of the PDMS membrane (Figures 2d-v). In the experimental validation of the deformation gradient, z-components were not considered, as they do not directly influence cell stimulation under the planar equibiaxial stretching. For further experimental characterization of the platform’s functionality and applicability, an inlet suction pression of −300 mbar was chosen, as it represented the maximum pressure corresponding to the maximum lowering of the PDMS membrane into the hollow cavity, applicable for this microfluidic platform configuration. Moreover, a suction pressure of −300 mbar corresponds to a strain range of 10% (Supplementary Figure S10c – average strain evaluation), which is consistent with the cardiac physiological range (Smiseth, 2018).

### 2.3 Simulative and experimental validation of mechanical stimulation-fluid flow interaction

**FIGURE 3:**
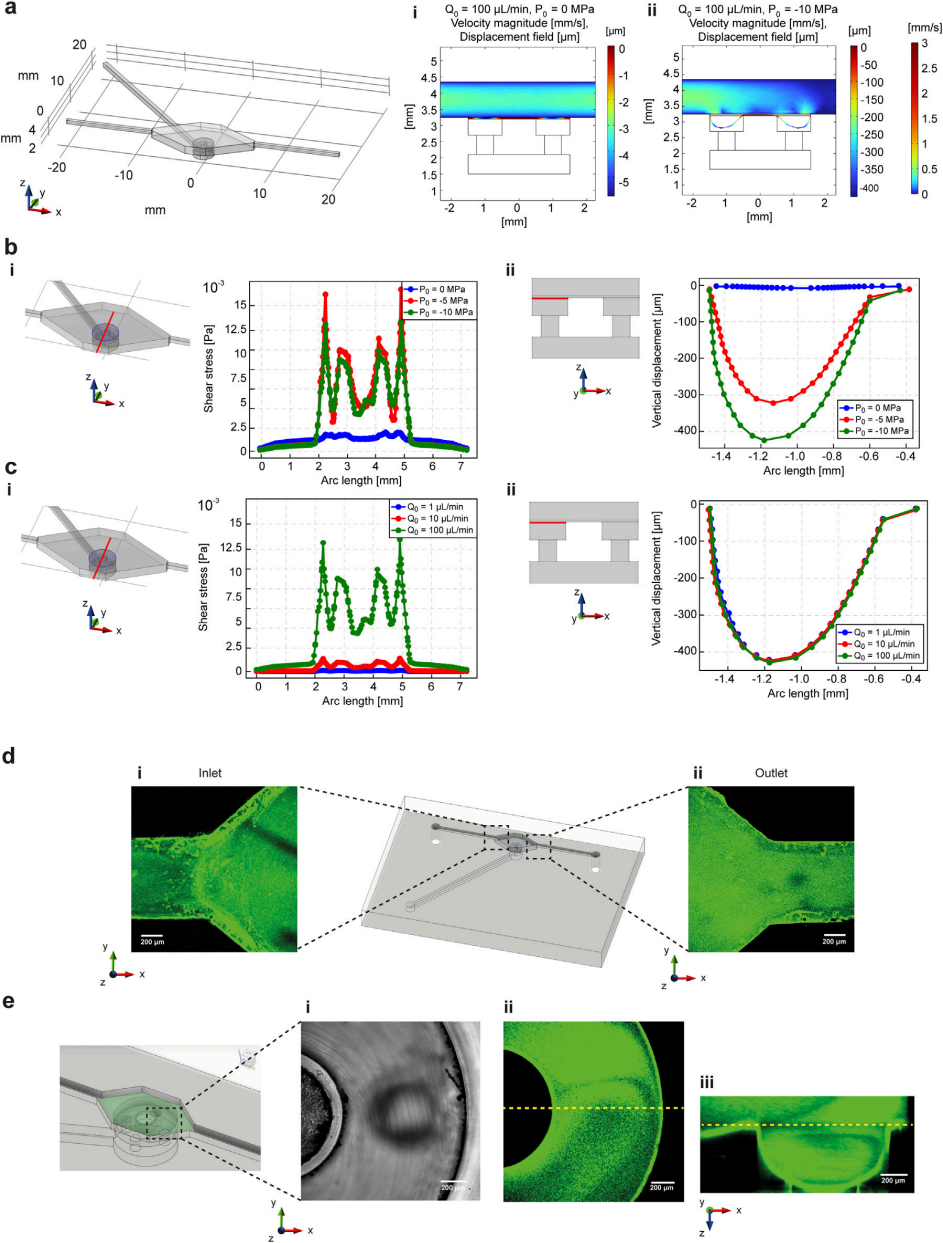
COMSOL simulations are compared to the experimental characterization of the biophysical and biochemical cues integration in the microfluidic platform. The simulations were performed to prove the effect of the combination of microfluidic fluid flow and deformation mechanics on the PDMS membrane. **(a)** COMSOL simulations were performed by coupling solid mechanics and fluid dynamics modules, considering the mechanical stimulation unit chambers and channels and one upper circuitry for the fluid flow of nutrients and biochemical substances. The effect of membrane deformation on the fluid flow was determined by changing the suction pressure at the inlet of the deformation channel (0 and −10 MPa) by keeping the inlet flow rate unchanged (100 μL/min). 3D simulations on a cross-section of the fluid flow and mechanical stimulation chambers demonstrated that the fluid flow velocity is affected by mechanical stimulation **(a-i, ii)**. In addition, fixed a cutline in the x-y plane corresponding to the fluid flow-PDMS membrane interface, shear stresses were evaluated **(b-i)** by changing the suction pressure for membrane deformation **(b-ii)**. The shear stresses changed but were limited to a range of low values. **(c)** The effect of the fluid flow on the deformation of the membrane was assessed by changing the inlet flow rate value (1, 10 and 100 μL/min) by keeping the suction pressure constant (−10 MPa). The simulations at the interface between the fluid flow and mechanical stimulation chambers **(c-i)** demonstrated that even if the shear stress is dependent on the flow rate changes, the vertical lowering was not affected **(c-ii)**. The experimental results confirmed the simulative characterization. **(d)** Flux of fluorescent nanobeads in water solution demonstrated that the fluid followed the shape of the chamber without bubble formation, both at the inlet **(i)** and outlet **(ii)** of the microfluidic platform. **(e)** A lateral cross section of the mechanical stimulation chamber demonstrated that the fluid flow of fluorescent nanobeads did not impair the vertical deformation of the PDMS membrane, as demonstrated by brightfield **(i)** and fluorescence **(ii)** images of the deformation chamber, and by the fluorescence reconstruction of the vertical lowering of the membrane **(iii)**.

Fluid flow was simulatively and experimentally validated in combination with mechanical stimulation to prove the ability of the microfluidic platform design to selectively combine both biophysical and biochemical stimuli. 3D COMSOL simulations were performed by combining the fluid flow chamber and channels with mechanical stimulation unit, described in paragraph 2.2 (Figure 3a). The effect of mechanical deformation on fluid flow was assessed by simulating the fluid velocity profile and PDMS membrane deformation at constant flow rate (100 μL/min) and variable air suction pressure (0, −5 and −10 MPa). The results showed that the velocity was affected by PDMS membrane deformation, as its lowering corresponded to an additional change in fluid volume (Figures 3a-i,ii; Supplementary Figure S8). However, the 1D velocity profile at different heights from the deformation area (Supplementary Figure S8b) and shear stresses at the fluid-membrane interface (Figure 3b-i) demonstrated that the parabolic velocity profile was unaffected and the highest values of shear stress were concentrated in the hollow cavity of the deformation chamber in which the membrane bends (Figure 3b-ii). Instead, in the central area of the plot (values of arc length between 3 and 4 mm), corresponding to the deformation pillar surface, shear stresses remained limited, meaning that cells will not be exposed to high shear stresses values when the fluid flow is working with the mechanical deformation. On the other hand, the influence of the fluid flow on the mechanical deformation of the PDMS membrane was assessed by varying the inlet flow rate (1, 10 and 100 μL/min) fixing the air suction pressure at the maximum value (−10 MPa). While the shear stress profiles increased proportionally to the inlet flow rate starting from 10 μL/min (Figure 3c-i), the PDMS membrane vertical lowering was not affected by the flow rate value (Figure 3c-ii).

Fluid flow was experimentally tested on the platform by flowing a water solution containing fluorescent nanobeads. As shown in Figure 3d, the shape of the microfluidic chamber effectively prevented bubble formation at both the inlet and outlet, confirming the fluid-dynamic simulation results regarding the shear stresses profile (Supplementary Figure S1). In addition, fluid flow was combined with mechanical stimulation of the PDMS membrane in the deformation chamber, demonstrating that membrane lowering into the hollow cavity occurred without the formation of bubbles or cavitation in the fluid flow (Figure 3e).

### 2.4 Topographical cues: micropattern characterization

**FIGURE 4:**
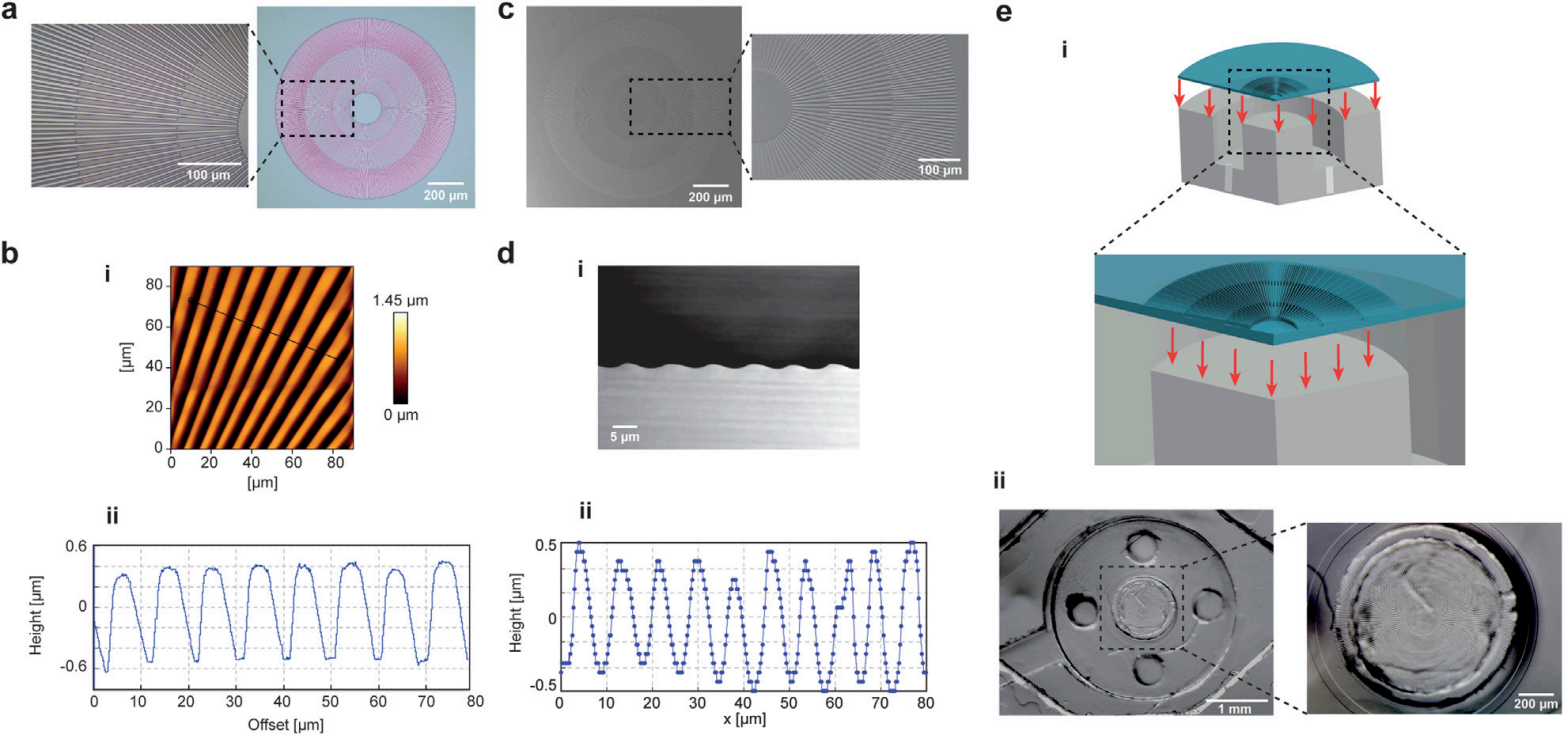
Micropattern characterization and integration in the microfluidic platform. **(a)** A micropatterned substrate was used to introduce topographic stimulation in the microfluidic platform. The topography radial arrangement is conceived to align the cells along the direction of the stretch. Images of the patterned master is reported, with a magnification on a sector of the micropattern to highlight the complexity of stripes arrangement to reproduce an ordered structure. **(b)** The master was characterized by AFM scanning. A patterned section of the master sample was scanned **(i)** and the depth quantification of the normalized line plot showed a height of 1 µm and a pitch distance of 10 µm **(ii)**. **(c)** Images of PDMS mold corresponding to the patterned master are reported. **(d)** The lateral cross-section of the PDMS mold was characterized by cryo-sectioning and SEM imaging **(i)** and the quantification of the line plot gave a pattern height of c.a. 0.9 µm with a sinusoidal-like waveform **(ii)**. **(e)** The PDMS flexible membrane was patterned and integrated in the microfluidic platform, so that the radial micropattern aligned with the circular central pillar of the mechanical stimulation unit **(i)**. A photograph of the membrane embedded in the device at the level of the deformation chamber is reported, with an inset showing the alignment of the micropattern to the underlying pillar **(ii)**.

A radial pattern was designed and fabricated using photolithography techniques to promote cellular alignment in the region subjected to equibiaxial mechanical stimulation (Supplementary Figure S11a). A representation of the patterned master is reported in Figure 4a. The characteristic pattern consisted of alternating ridges and grooves, with maximum and minimum widths of 5 μm and 2 μm, respectively. The radial symmetry of the structure dictated the alternating arrangement of the stripes, and a uniform organization was maintained along the center of the symmetry, dividing the pattern into sectors along the radial direction. The topography dimensions were designed to precisely fit the external edges of the circular pillar in the platform’s deformation unit. The thickness of the stripes was controlled by photoresist spin coating and characterized using AFM scanning (Figure 4b-i). Depth quantification of the normalized line plot revealed that the micropatterned photoresist master had a height of 1 μm and a characteristic square profile (Figure 4b-ii). The micropatterned master was fabricated on a flexible substrate to facilitate handling during the alignment and peel-off of the thin PDMS membrane prior to bonding within the microfluidic device (Supplementary Figure S11b). Uncrosslinked PDMS was poured, spin-coated, and cured on the master’s surface to obtain the thin patterned membrane (Figure 4c). SEM imaging was used to characterize the morphology and dimensions of the micropatterned PDMS mold (Figure 4d; Supplementary Figure S11c). The front view of the micropattern revealed that the radial stripes were accurately reproduced during the molding step, with stripe widths close to 5 µm (Supplementary Figure S11c-i). The height of the patterned PDMS was determined by cryo-sectioning and SEM imaging of the membrane’s side view (Figure 4d-i; Supplementary Figure S11c-i). The images showed that a sinusoidal profile formed after polymerization of the uncured PDMS over the photoresist master. FIB-SEM profiling revealed a slight reduction in pattern height (from 1 μm to 0.9 µm), whereas the peak-to-peak distance of 10 µm confirmed the characteristic dimensions of the micropatterned master, consistent with AFM characterization (Figure 4d-ii).

Finally, the micropatterned PDMS membrane was integrated into the microfluidic platform (Supplementary Figure S11b; Figure 4e-i). The circular pattern was aligned with the boundaries of the pillar in the mechanical deformation unit, allowing the deformation field to follow the orientation of the pattern. Stereomicroscope images of the device-integrated micropatterned PDMS membrane (Figure 4e-ii) showed accurate alignment of the radial pattern with the underlying circular surface of the pillar.

### 2.5 HL-1 cardiac cell line seeding and viability in the microfluidic platform

HL-1 CMs were seeded on the stimulation platform after functionalization with ECM proteins to enhance the compatibility of the PDMS substrate for cell adhesion and survival (Supplementary Figure S12). Fibronectin was selected as the ECM protein for HL-1 attachment (Choi et al., 2013). To determine which fibronectin concentration yielded the highest cell survival and proliferation, three different concentrations (1, 10 and 100 μg/mL) were tested over 72 h (Supplementary Figures S11b,c). Fibronectin concentrations of 10 and 100 μg/mL significantly improved HL-1 proliferation on PDMS, with doubling cell count after 72 h and 24 h after seeding, respectively. Given the physiological behavior of HL-1 cells in conventional culture plates, where the cell doubling time is c.a. 48 h, an intermediate fibronectin concentration of 50 μg/mL was chosen to optimally enhance HL-1 cell adhesion to PDMS substrates.

**FIGURE 5:**
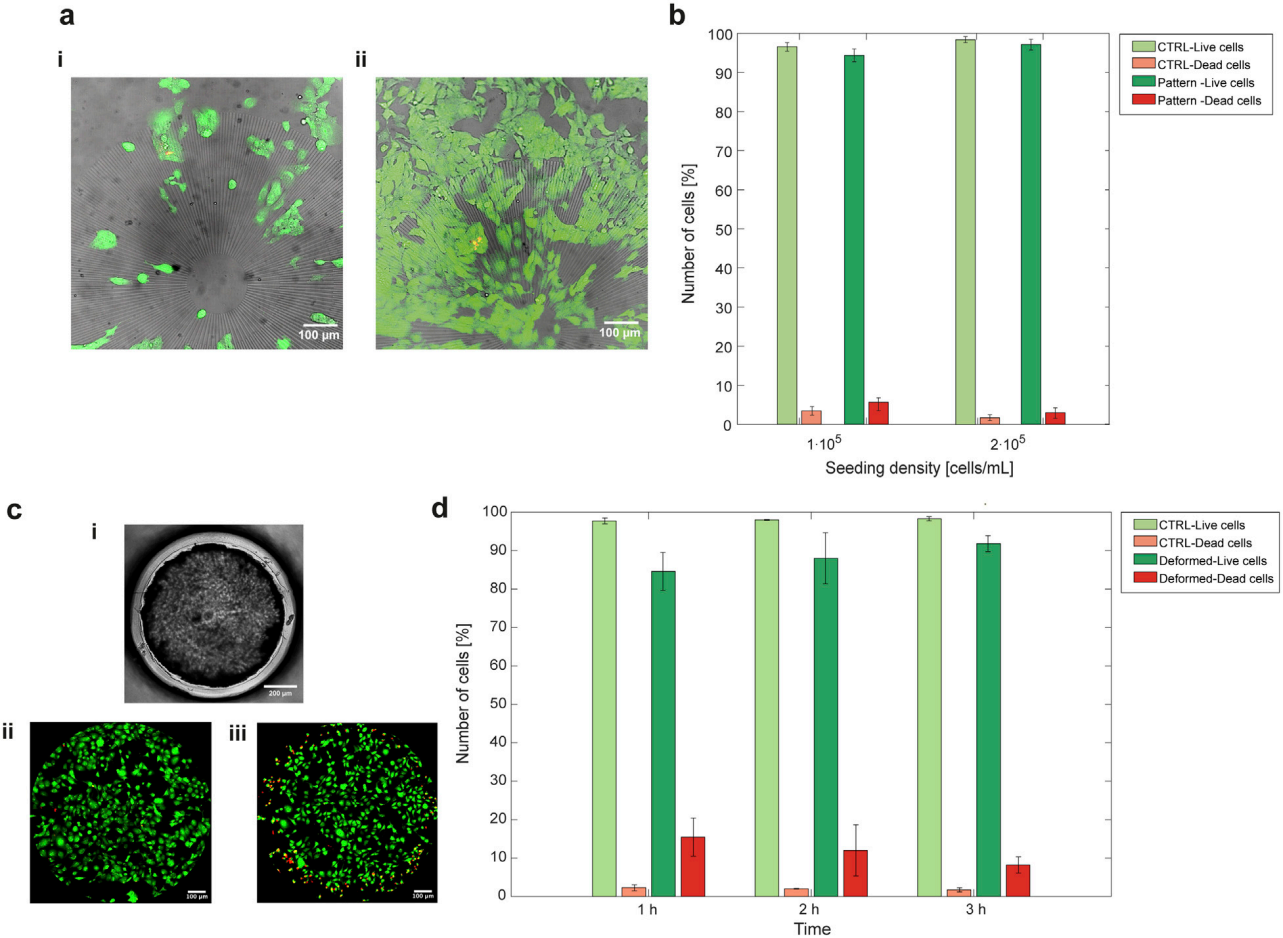
HL-1 cardiac cells proliferate and survive when seeded on platform-embedded PDMS membrane, with or without surface patterning and after prolonged mechanical stimulation. **(a)** HL-1 cells were seeded on patterned PDMS at two seeding densities, 1⋅10^5^ cells/mL **(i)** and 2⋅10^5^ cells/mL **(ii)**. In brightfield there is the micropattern, live cells stained with Calcein AM (green) and dead cells with Propidium Iodide (PI) (red). **(b)** The quantification of percentage of live cells over dead cells on flat PDMS (CTRL) and patterned PDMS, seeded with a density of 1⋅10^5^ cells/mL and 2⋅10^5^ cells/mL, reported that, in all the four cases, cell viability was supported over cell mortality. **(c)** HL-1 cells viability was also assessed after mechanical deformation. Cells were seeded on flat PDMS embedded in the microfluidic platform (brightfield image in **c-i**) and undeformed cells survival **(c-ii)** was compared to that of cells undergoing equibiaxial deformation **(c-iii)**. **(d)** Live-dead quantification demonstrated that vitality is slightly affected at the beginning of the deformation (after 1 h), but a vital cell culture is still present upon mechanical deformation. One-way ANOVA statistical analysis revealed non-significant differences between the groups in both live-dead assay quantifications. The values of live and dead cells in the plots are represented as mean ± SEM.

Cell survival was assessed using a live-dead assay, where HL-1 cells were stained to determine the percentage of vital versus dead cells after interaction with the PDMS substrate. The assay on flat substrate demonstrated the platform’s ability to support cell survival following fibronectin functionalization and 24 h of seeding (Figure 5b – CTRL samples). In addition, the live-dead assay was repeated at different cell seeding densities (Figure 5a). The results showed that cell viability remained unaffected, even at half the seeding density. For comparison, HL-1 survival was also tested on patterned PDMS after 24 h of seeding, and the enhanced predominance of viable cells over dead cells was evident on the patterned PDMS as well (Figure 5b–Pattern samples).

The effect of equibiaxial mechanical deformation on cell survival was characterized among 1 h, 2 h and 3 h of continuous deformation in the microfluidic platform (Figure 5c). The results showed an increase in death cells relative to live cells within the first hour of deformation, followed by slightly higher cell survival after 3 h of mechanical stretching (Figure 5d).

### 2.6 HL-1 cardiac cell line alignment to the micropatterned substrate

**FIGURE 6:**
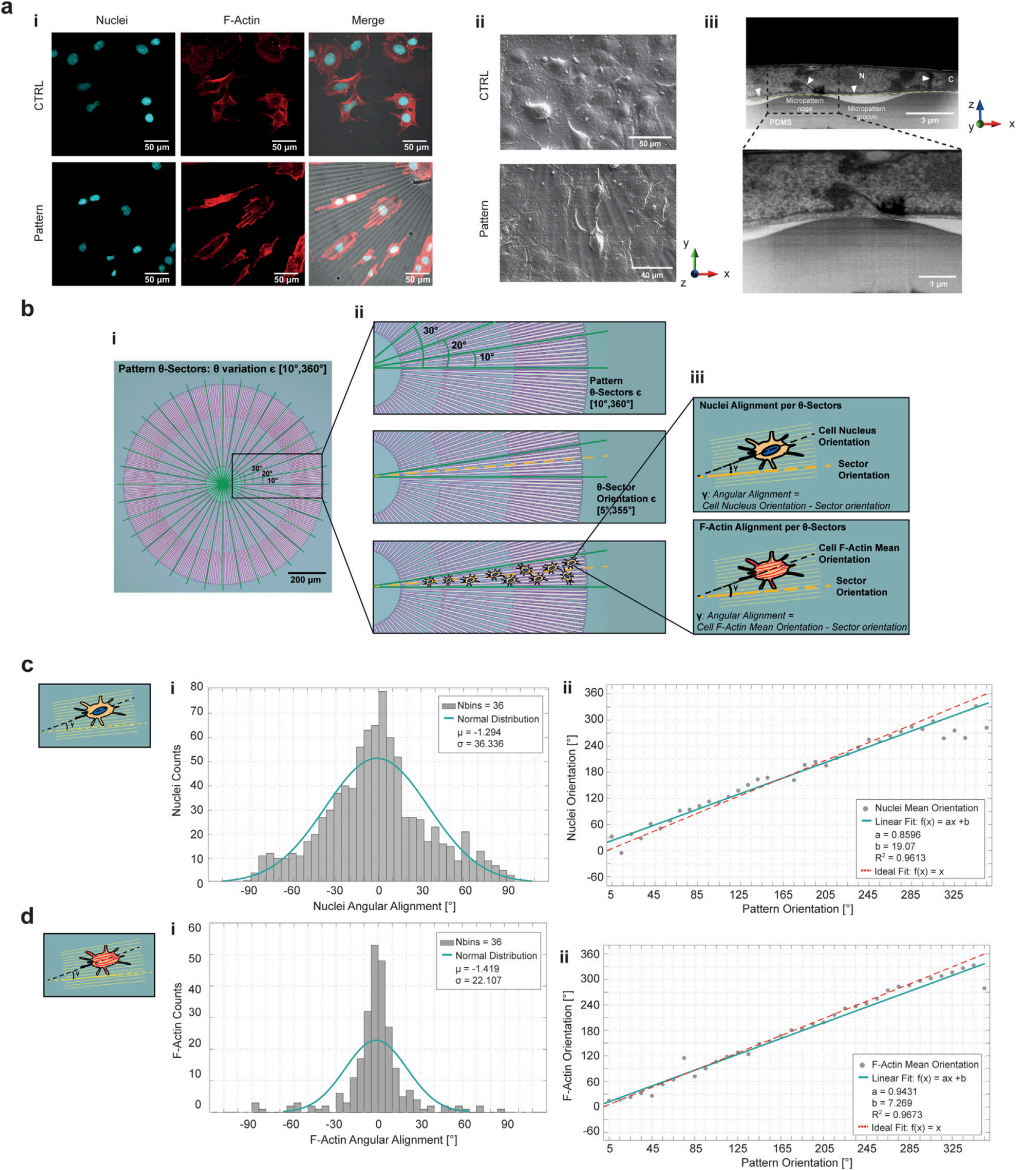
HL-1 cardiac cells aligned to the radial micropattern. **(a-i)** Confocal images of HL-1 cardiac cell line seeded on flat PDMS membrane as control sample (top row) and on micropatterned PDMS membrane (bottom row) to compare the alignment capability of the topography to a non-patterned surface. The pattern was represented in brightfield, nuclei were stained with cyan and actin fibers in red. **(a-ii)** SEM images of HL-1 cells seeded on control sample (top panel) and patterned PDMS (bottom panel). **(a-iii)** FIB-SEM cross-sectioning of a HL-1 cell seeded on the micropatterned PDMS. It is possible to recognize the nucleus (N), cytoplasm (C) and the nuclear membrane (white arrows). The cell-PDMS boundary is highlighted to empathize the cell arrangement over the pattern ridge (yellow dotted line). An inset on cell-PDMS ridge interaction point is reported. **(b)** Cell alignment logic for the radial micropattern. The pattern was divided into sectors of 10° **(i)**, in each sector cell nuclei and f-actin orientation were quantified **(ii)**. The angular alignment of nuclei and f-actin was computed as the difference between nuclei and f-actin orientation and sector orientation, which is the mean angle of the selected sector **(iii)**. Histograms of nuclei **(c-i)** and f-actin **(d-i)** angular alignment are representative of the deviation of cells orientation with respect to the radial micropattern. The histograms peak around 0°, meaning that cells are oriented along the topography. Linear fitting of nuclei **(c-ii)** and f-actin **(d-ii)** orientation in pattern orientation demonstrated that there is a univocal correspondence between nuclei/actin fibers and micropattern arrangement.

A comparison of f-actin and nuclei fluorescence with SEM images of HL-1 cells cultured for 24 h on patterned and non-patterned PDMS revealed a clear difference in cell orientation, organization and shape (Figure 6a-i,ii; Supplementary Figures S13a,b). Non-patterned (flat) PDMS, exhibited a random distribution of actin fibers throughout the observation area, while patterned PDMS produced an ordered and highly aligned arrangement of cells that conformed to the imposed topography. FIB-SEM cross sections were acquired to characterize the arrangement of cells with respect to the micropattern profile. In Figure 6a-iii, the FIB-SEM cross section of a cell seeded for 24 h on top of the patterned PDMS demonstrates that the CM outlines, which interacted with the PDMS surface and are represented as a dotted yellow line, extended from ridge to ridge of the micropattern, avoiding entering the groove area. For further characterization, a circle was defined at the outer boundary of the pattern, and a polar coordinate system was centered at the circle’s midpoint, as shown in Figure 6b. Sectors of 10° were isolated by varying the θ direction of the polar coordinates (Figures 6b-i,ii). Within each θ-sector, cell orientation relative to the pattern was precisely measured by detecting the tilt of nuclei and f-actin with respect to the stripes of the PDMS ridges and grooves. The alignment of nuclei and f-actin within each θ-sector was calculated as angular alignment (**γ**), defined as the difference between the cell nucleus orientation and the sector orientation for nuclei angular alignment, and as the difference between the f-actin mean orientation of individual cells and the sector orientation for actin filaments angular alignment (Figure 6b-iii). The histograms of nuclei and f-actin angular alignment across the cell population were fitted with a normal distribution with a mean (μ) approaching 0 (Figures 6c-i, d-i). In addition, the plots of mean orientation of nuclei and f-actin versus the mean orientation of pattern in each θ-sector were well represented by a linear model, with slope approaching 1 (Figures 6c-ii, d-ii). The effect of deformation gradient was quantified by diving the radial micropattern along the r-direction in three areas corresponding to the main level of strains distribution (Supplementary Figure S13). In this way nuclei mean orientation was compared to the orientation of the micropattern in each θ-sector under equibiaxial deformation. The alignment of the nuclei was not affected by the deformation gradient, since the histograms of angular alignment peaked around 0 and the comparison between mean orientation of micropattern and nuclei is fitted by a linear model in each r-sectors.

### 2.7 HL-1 cardiac cell line response to equibiaxial mechanical deformation and micropattern

**FIGURE 7:**
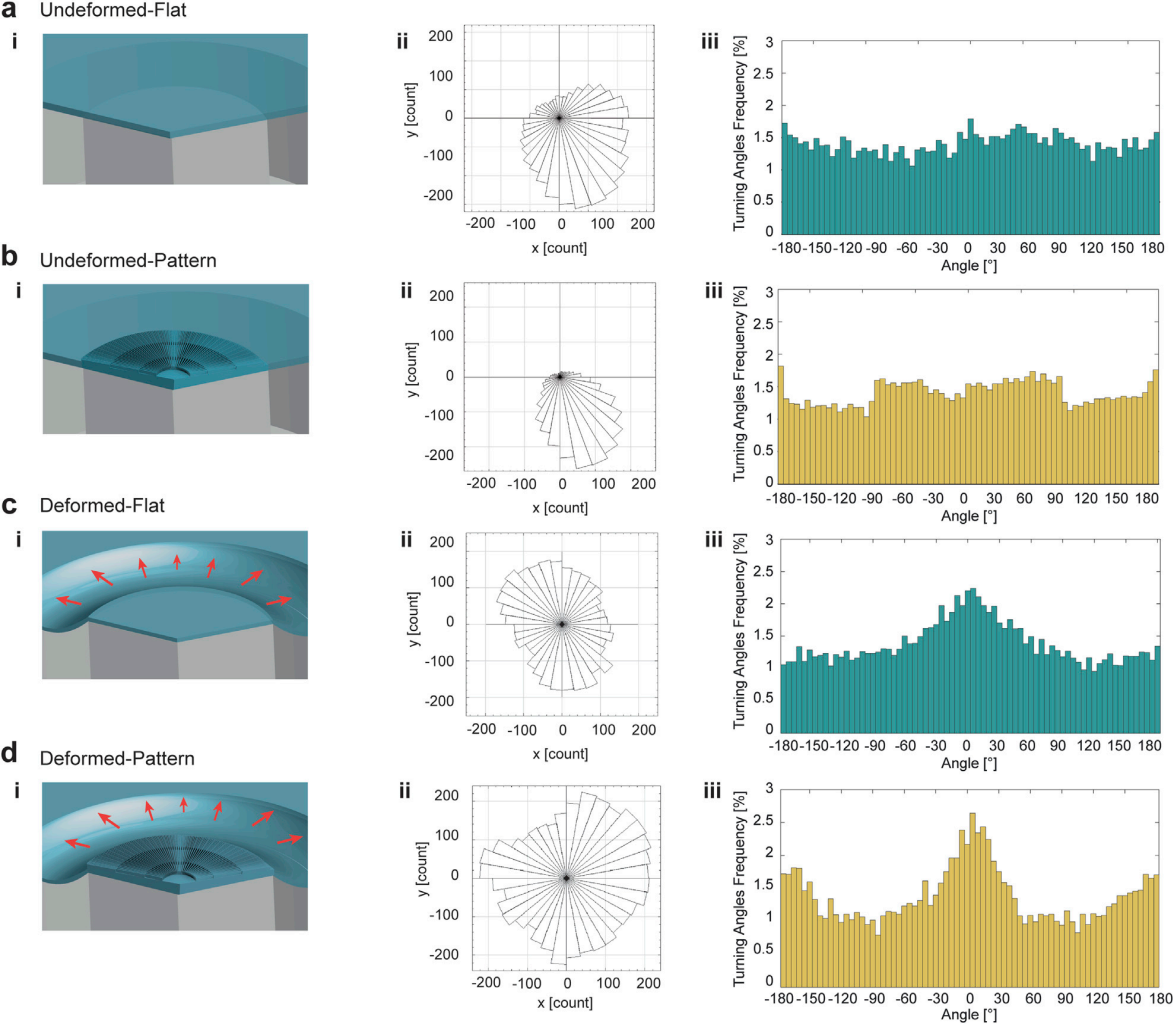
HL-1 cardiac cells migration response to mechanical deformation and the topography. HL-1 cells were tracked for 1 h in the platform when they were seeded on undeformed flat substrate **(a)**, undeformed patterned substrate **(b)**, deformed flat substrate **(c)** and deformed patterned substrate **(d)**. Schematics of the configurations are reported in column **(i)**. Rose plots of cells tracked for 1 h demonstrated a random distribution for undeformed cell populations **(a,b-ii)**, turned to a more uniform arrangement of trajectories for 1 h equibiaxially deformed cell population **(c,d-ii)**. In particular, the presence of the micropattern combined to the equibiaxial deformation accentuated the uniformity of the trajectories distribution in space **(d-ii)**. Turning angles distributions of cells trajectories tracked for 1 h represent the migration type of cells in response to the microenvironment signals. Cells moved persistently when they were equibiaxially deformed **(c-iii)** and even more when the mechanical deformation is applied to patterned cells **(d-iii)**.

The impact of equibiaxial deformation on CMs was evaluated by quantifying their migratory ability and morphology variations on the platform, since cell migration is essential in the process of adaptation to external conditions, as discussed in paragraph 3. Mechanical stretching of the cells seeding substrate was applied and sustained for 1 h, after 24 h of cell cultivation. Afterward, the trajectories of HL-1 cell nuclei were tracked by recording their positions every minute on the surface of the pillar. The experiment was performed on both flat and patterned PDMS substrates embedded in the platform (Figures 7a,b,c,d-i). The tracks of moving cells were plotted as trajectory occurrences in the x-y plane. The results revealed a significant difference in the distribution of cell trajectories between deformed and undeformed substrates (Figures 7a,b,c,d-ii). On undeformed flat and patterned PDMS substrates, HL-1 cell population tracks tended to orient towards one direction (Figures 7a,b-ii; Supplementary Figure S14b). In contrast, deformed flat and patterned PDMS substrates promoted uniformly distributed orientation of cell population trajectories above the circular pillar surface (Figures 7c,d-ii; Supplementary Figure S14b).

Histograms of turning angles for flat and patterned PDMS showed that, in the absence of deformation, HL-1 cells exhibited random motion (Figures 7a,b-iii). Deformation induced turning angles distributions that peaked around 0° (Figures 7c,d-iii). In the motion analysis, the response of HL-1 cells to mechanical deformation was further evaluated by examining their migration velocity (Supplementary Figure S14c), with higher and narrower distribution for velocity of cells moving on deformed patterned substrates. Cell body spreading quantification also demonstrated a reduction in cell area when mechanical stimulation was applied, with a slight reduction in polarity for both flat and patterned samples (Supplementary Figure S15).

## 3 Discussion

The development of *in vitro* platforms that accurately recapitulate the complex microenvironmental conditions experienced by cells remains a major challenge in tissue engineering and mechanobiology studies. This is particularly valid in the case of cardiac microenvironment stimuli and their complex interplay. In this study, we present a novel microfluidic device that simultaneously integrates mechanical stimulation, topographical cues and biochemical flux within a highly controllable system of microchannels and micropneumatic actuation. By enabling precise spatial and temporal regulation of these key factors, the platform provides a biomimetic environment capable of reproducing native cardiac tissue conditions while maintaining simplicity, making it a relevant case study.

The proposed setup required a multi-layered structure to separately deliver air vacuum for mechanical stimulation and culture medium for cell seeding and feeding. Furthermore, materials must withstand the mechanical stretching load while ensuring biocompatibility and high structural organization to support proper cell proliferation and survival. PDMS has been favored in early lab-on-a-chip models, especially in the case of cardiac tissue reproduction, due to its affordability, ease of fabrication, transparency with consequent optical accessibility, controllable mechanical properties, sterilizability, oxygen permeability, and compatibility with surface modifications (Akther et al., 2020; Miranda et al., 2021). In the proposed platform, PDMS was used for its flexibility to create a stretchable substrate for cells seeding and mechanical stimulation, whereas PMMA, a tough, lightweight thermoplastic, was chosen for the platform. Indeed, PMMA allows for the multi-layer implementation while maintaining the shape and integrity of channels and chambers (Faghih and Sharp, 2018). Additionally, its transparency provides optical accessibility to real-time cell culture imaging with standard microscopy instruments. Beyond the efficiency of commercially available mechanical loading systems, the proposed platform leverages the key advantages of microfluidic technologies, such as the miniaturization of the mechanical stimulation at the cell scale, as well as the reduction of the handling volumes for both culture medium flow and air suction, which not only offers an economic advantage but also facilitates the integration of biophysical and biochemical cues. Additionally, the microfabrication techniques used for the platform can be easily integrated with photolithography, enabling the inclusion of high-resolution micropatterned structures and allowing the efficient combination of different microenvironmental cues within the same chamber.

The platform architecture allows simultaneous pneumatic actuation and nutrient exchange while maintaining the independence of the applied cues, as validated through simulations and experimental performance of the platform. The device’s design, featuring a thin PDMS membrane stretched across a central rigid pillar, generates controlled equibiaxial mechanical stimulation. In the *in vivo* context, isotropic stretch does not reflect the mechanical constraints that cells physiologically experience in living tissues, which are typically direction-dependent and related to anisotropic expansions during the interaction with their surroundings (Wheatley, 2020; Friedrich et al., 2019). In the *in vitro* modeling of the cellular microenvironment, although uniaxial loading systems provide detailed characterizations of cellular response to directional mechanical stimuli, they do not adequately replicate the complex strain environments found in native tissues, in which cells may be subjected to distinct strain orientations. In this direction, our platform, introducing a radial configuration, is also able to generate a strain gradient across the substrate emulating the mechanical heterogeneity observed in the physiological microenvironment. The characterization of principal stresses and strains provided insight into material deformation, specifically indicating the extent of stretching or compression under the applied load (Rubin and Krempl, 2010). In particular, simulations confirmed that the deformation occurred through pure stretching without rotational or shear components, also validated experimentally by tracking fluorescent nanobeads embedded in the PDMS substrate. Moreover, the microfluidic combination of mechanical deformation with nutrient flux was simulatively characterized. The results demonstrated that although fluid flow locally changed upon mechanical deformation, shear stresses remained limited to a range of low values, in the order of mPa, compatible with the physiological conditions for cell culture in which a negligible stimulation can be attributed to shear stress forces (Boycott et al., 2013; Espina et al., 2023). Conversely, mechanical stimulation was not affected by the fluid flow introduction, even at higher values of inlet flow rate. These findings indicate that the flux of substances and the biophysical stimuli can be applied simultaneously with the designed configuration, without altering the individual effects of each stimulus. This result underscores the high versatility of the platform in simultaneously replicating various cell microenvironmental stimuli.

To test the device’s ability to support cell survival, viability assays were performed by changing HL-1 cell seeding density on the flat and patterned PDMS substrates, as well as with and without the mechanical deformation. The highest seeding density was chosen as the most appropriate, as it enhanced cell-cell communication while preventing full confluence of the cell layer. This approach was adopted to exclude cell-cell contact as an additional variable in the cell response analysis while emphasizing the role of the micropattern and mechanical stimulation as key influencing factors. Static mechanical stretch was applied, and the cells’ viability was evaluated. Although dynamic stretching is more physiologically relevant for mammalian cells applications, and particularly for CMs, a static stimulation was chosen as proof of concept to test the platform functionality and the interaction of cells with the introduced stimulation. Mechanical deformation in the first hour of stimulation induced an initial increase in HL-1 cell death but only localized at regions of high stress concentration, noticeably limited to the boundary of the pillar, where stresses increased due to the extension and bending of the PDMS membrane over the pillar walls (Supplementary Figures S4b,c). These cell viability results support the platform’s suitability for further studies on the effect of long-term mechanical deformation on the cells behavior (Ronaldson-Bouchard et al., 2018; Han et al., 2020; Molnar and Labouesse, 2021).

In addition to the previously discussed rationale, this study aimed to address a major limitation of standard *in vitro* models, where cells are often randomly arranged, resulting in heterogeneous response to microenvironmental stimulation, in particular to the mechanical cues. In native tissues, cells exhibit highly ordered architectures and their orientation relative to the deformation direction influences mechanotrasduction pathways in a critical way (Dalby et al., 2014; Vining and Mooney, 2017). Standardizing cell response to mechanical stimulation requires introducing a precise spatial organization of cells, which can be controlled by tuning the topography of the cell seeding substrate. In particular, topographical cues from the tissue ECM guide the orientation and alignment, especially in cardiac cells, in which they are critical for efficient heart contraction and conduction of the action potentials (Parker and Ingber, 2007). On the microscale, features comparable to the size of a cell can influence its alignment and elongation through contact guidance (Parker and Ingber, 2007; Guvendiren and Burdick, 2013; Dalby et al., 2014; Ahn et al., 2023). Filopodia, which act as the cell’s sensing machinery for material surfaces, are composed of actin filaments typically about 5 μm in length (Schäfer et al., 2011). Surface features extending above this length may not be easily detected by filopodia. After adhesion, cells start to conform to their topographical microenvironment by forming stable focal adhesion (FA) sites on the substrate. Microscale patterns can guide cell morphology and cytoskeletal rearrangement, provided that the dimensions of the topography are smaller than the characteristic size of FAs, which, at full maturation stage, remains below 10 μm (Teixeira et al., 2006; Nguyen et al., 2016; Tabdanov et al., 2018; Buskermolen et al., 2020). Ventre et al. demonstrated that cells aligned and migrated along the direction of ridges and grooves with a characteristic dimension of 5 μm, exhibiting coherent alignment of actin fibers influenced by pattern geometry (Ventre et al., 2014). In addition, previous research has shown that the formation of FAs on surface protrusions with height lower that 1 μm discourages the formation of complexes in the pits of the structures, confining the alignment of the cells on the top ridges (Seo et al., 2013). These findings support the working principle of our radial topography, as the dimensioning of the micropattern width of 5 μm and height of around 1 μm promoted strong alignment of HL-1 cells on the top of ridges stripes, which guide cell elongated arrangement at both cytoskeletal and filopodial levels, mediated by mechanotransduction phenomena. Moreover, a radial arrangement of the micropattern was chosen for the first time to orient and homogenize cells towards the equibiaxial mechanical stimulation direction. The topography effectively directed HL-1 cell alignment, as confirmed through nuclear and actin filament orientation analysis. These results demonstrated that the radial pattern successfully aligned both nuclei and actin filaments, as their average orientation closely matched the micropattern sector orientation. Therefore, to further verify the alignment capability of the design topography, the mean orientation of nuclei and f-actin in each θ-sector was plotted against the mean orientation of the corresponding θ-sector, demonstrating a clear correspondence between cell’s mean orientation and pattern’s mean orientation in each θ-sector. These outcomes collectively indicate that the proposed pattern effectively aligned HL-1 CMs in terms of both nuclei and f-actin, key components in mechanotransduction pathways. Moreover, due to the deformation gradient arising on the surface of the deformation pillar, a question arose regarding whether cell alignment shifted in response to the deformation-induced gradient. To address this, a quantification of nuclei angular alignment histogram was performed on the three sectors identified by the gradient arising from deformations map along the r-direction (Supplementary Figure S13). The results demonstrated that the gradient logic did not affect cells’ alignment, which consistently followed the micropattern direction without any difference in the three sectors.

Cell migration is an essential mechanism that cells adopt to respond to the surroundings. For this reason, it is commonly investigated in the literature to explain how cells react when an external stimulus is applied (Gonnerman et al., 2012; Yamada and Sixt, 2019; Hu et al., 2021; Merino-Casallo et al., 2022). In the current study, the distribution of cell trajectories revealed distinct behaviors between HL-1 cells seeded on deformed and undeformed substrates within a 1 h observation period (Figures 7a,b,c,d-ii). To date, a short observation window was considered for the migration experiment to enable a fundamental characterization of early-stage cells’ response to biophysical cues, as typical migration rates for slow-moving cells can be approximated to one cell-body length per hour (Ge et al., 2020; Guetta-Terrier et al., 2015). At short observation times, it is also possible to capture the early mechanotrasduction response, including focal adhesion dynamics, actin and cell polarization. The results showed that, in the absence of deformation, the tracks tended to orient in a single direction, with cells randomly moving. This was likely due to collective cell movement, driven by cell-cell interaction rather than external guidance (Shellard and Mayor, 2020). The influence of the deformation promoted a uniform distribution of cells trajectories. Notably, cells on deformed patterned PDMS exhibited the most uniform distribution of trajectories in the x-y migration plane. The radial symmetry of the patterned PDMS influenced the distribution of cell trajectories, but only if it was combined with the equibiaxial mechanical deformation, indicating that the pattern alone could not direct HL-1 cells movement. Further quantification of HL-1 migration under mechanical deformation was performed by analyzing the turning angles distribution, which provided an indication of the persistence of cell motion (Supplementary Figure S14a). The histograms showed that deformation increased persistence in cell migration. Patterned deformed substrates induced a more pronounced and narrower peak of turning angles compared to flat deformed substrates, with statistically significant differences between all the conditions (p values <0.05 for all the distributions, Supplementary Table S4, Supplementary Material), confirming that the combination of mechanical and topographic cues guides and directs cardiac cells movement. The velocity distribution of HL-1 cells peaked at higher velocities, with a narrower spread, for cells migrating on radially patterned PDMS after 1 h of deformation. This suggests that mechanical deformation of patterned substrates not only guides cell movement orientation but also significantly influences migration speed. Moreover, morphological quantifications on cell area and polarity exhibited that HL-1 cells seeded on undeformed flat substrates exhibited less spreading with respect to the patterned counterpart, which instead showed higher polarity. However, at early stage of equibiaxial mechanical stimulation, this equilibrium was disrupted, resulting in reduced spreading and polarity for both flat and patterned samples. This is consistent with the migration data results, in which the arrangement of the trajectories’ direction shifted from unidirectional to multidirectional when the equibiaxial mechanical stimulation was applied, resulting in a dynamic rearrangement of the cells. These findings can be explained by the role of mechanical stress on cell integrins, which play a key role in activating focal adhesion kinase (FAK) and factors like the Rho GTPase family, known to enhance cell migration by promoting forward elongation and posterior contraction (Raftopoulou and Hall, 2004; Gardel et al., 2010). For instance, in a study by Takemoto et al., mechanical stimulation in the form of static and cyclic stretch increased both migration velocity and cell alignment compared to non-stretched cells (Takemoto et al., 2023). Moreover, previous literature supports the role of patterning in enhancing migration persistence and velocity. Micropattern with characteristic feature of 5 μm or larger can guide the directed cell migration (Ventre et al., 2014; Nam et al., 2016). Additionally, Slater et al. found that in endothelial cells, restricting the size of adhesion sites resulted in smaller, more dynamic adhesion sites, which enhanced cell movement by bypassing the slower process of disassembling larger and mature adhesions (Slater et al., 2015). This may explain the faster migration observed on patterned PDMS compared to flat substrates, as well as the exclusion of focal adhesion formation in the pattern grooves, which can otherwise slow the migration process. In our study, the integration of a radial micropattern with equibiaxial deformation demonstrated an enhanced and homogeneous cell alignment across the deformation substrate and a coherent cell response to the mechanical stimulation. The combination of the mechanical deformation with a controlled topographic micropatterning of the cells is crucial to achieve a uniform and homogeneous response of the stimulated cells. By directing actin organization, the combination of equi-biaxial deformation and micropatterning effectively conditions downstream processes related to mechanotransduction, including the anisotropic organizations of actin fibers and coordinated migration of the cell body. Based on the observed behavior, it is reasonable to suggest that the mechanical stimulation alone activates integrin sensing and FAK-Rho pathways, ultimately leading to cytoskeletal rearrangement and forward movement in the direction of stimulation. The introduction of topographic contact guidance enhances this effect, restricting cell adherence to the ridges with smaller and more dynamic adhesion sites, leading to a faster migration outcome. The redistribution of the trajectories is enhanced when micropattern is coupled to mechanical stimulation, meaning that the mechanical stimulation is the main driver of migratory behavior, while the coupling of two biophysical cues, i.e., stretch and topography, further modulate and enhance cell arrangement.

Previous studies have integrated mechanical stimulation and topographies to modulate cardiac cells behavior to better mimic their physiological conditions. Kim et al. showed that the combination of grooved PDMS and tensile uniaxial stress improved CMs maturation and modulated sarcomeres orientation orthogonally to the micropattern direction (Kim et al., 2023). Similarly, Navaee et al. took a step forward in recapitulating the 3D helicoidal organization of the ventricular architecture, achieved by combining opposing mechanical and topographical stimulation, which led to cells tilting and enhanced maturation (Navaee et al., 2023). Despite the promising advances of these works, uniaxial deformations remain limited in capturing the mechanical complexity of cardiac tissue and lack a comprehensive explanation on mechanotrasduction pathways involved in the proposed models. With our platform we introduce a complex mechanical stimulation in the form of equibiaxial stretch, along with radial spatial organization which enables homogeneous mechanotransduction responses, better characterized in terms of cells’ alignment and migratory behavior. Siddique et al. applied an equibiaxial mechanical stimulus to enhance CMs maturation, but along with parallel anisotropic ridges and grooves and it was not integrated in a microfluidic device (Siddique et al., 2022). Also in this case, although the results are crucial for elucidating cell response, this spatial arrangement of CMs fails to reproduce physiological conditions. Advantageously, our platform offers a novel perspective by delivering combined stimulations in a microfluidic-integrated context, implementing both biophysical and biochemical cues through microactuation and microchannel design. The proposed model benefits from the advantages of microfluidic applications within a miniaturized, controllable environment. To our knowledge, this is the first demonstration of such an integrated approach, capable of interfacing biophysical cues with biochemical stimuli in a complete cell microenvironment.

## 4 Conclusion

Overall, we present a microfluidic platform that enables the stimulation of cells with multiple microenvironmental signals, ranging from fluid flow to biophysical cues, in a comprehensive yet simple and easily assembled system. The chosen design allowed the integration of fluid flow, mechanical stimulation and topographical cues into an efficient configuration, in which each signal’s functionality can be individually modulated while simultaneously exploiting the key advantages of the microfluidic approach. In the application reported herein, this combination of signals helped demonstrate a synergistic effect of mechanical stimuli and pattern-induced alignment on cardiac cell response. The chosen radial pattern was particularly useful to obtain a homogeneous cell arrangement, while the application of equibiaxial mechanical stretch ensured a uniform stimulation of the cells. This offers valuable insights into cell behavior control, particularly in the activation of mechanobiological processes essential for various tissue types. This bioengineering approach, which strategically integrates microenvironmental stimuli in a microfluidic context, underscores the importance of accurate recapitulation and control of the cell microenvironment *in vitro*. The platform’s ability to precisely control mechanical deformation enables the replication of the previous tests under cyclic deformation. This feature is crucial, as cyclic mechanical stimulation has been shown to enhance the physiological response of various cell lines, including cardiac cells (Marsano et al., 2016; Nitsan et al., 2016). The introduction of a microfluidic circuitry for fluid flow, combined with mechanical and topographical cues, paves the way for further studies aimed at implementing pathological models. Consequently, this platform can serve as a valuable tool for testing drugs and treatments to assess the effects of healing strategies on cell cultures within a comprehensive microenvironmental context.

## 5 Materials and methods

### 5.1 Microfluidic platform fabrication

The device was designed in AutoCAD (Autodesk), with both 2D models for fabrication and 3D models for the COMSOL Multiphysics simulations. The microfluidic device was fabricated using a micro-milling machine (Minitech Machinery Corp.), to remove material and retrieve the platform microchannels and chambers from a bulk poly (methyl methacrylate) (PMMA) piece. Additional specifications regarding the characteristic dimensions of the microfluidic platform chambers and channels are reported in Figure 1b. Individual layers of PMMA were milled to create the channels and chambers, and these layers were then bonded together to assemble the microfluidics apparatus. Consecutive PMMA-PMMA layers were bonded by dissolving a thin superficial layer of the material at the interface using a solvent-based approach adapted from Bamshad et al. (2016) Isopropyl alcohol (IPA) was used as the solvent for PMMA plasticization at 68 °C. The bonding surfaces were washed in pure IPA to remove any residues that could impair adhesion between the layers. A 70% IPA solution in deionized water was then poured on the surface of one PMMA layer, which was brought into contact with the other layer for bonding. After precisely aligning the layers, clamps were used to press them together, improving material integration at the interface. Following 10 min at 68 °C, the layers were allowed to cool to room temperature.

A deformable membrane was fabricated and integrated into the microfluidic device to complete the microfluidics-driven mechanical stimulation unit. Polydimethylsiloxane (PDMS) was chosen for its adaptable mechanical properties, which can be adjusted by varying the prepolymer-to-curing agent ratio. A 20:1 ratio was selected to increase the flexibility of the PDMS membrane under deformation. PDMS (Sylgard 184, Dow Corning, MI, USA) was degassed to remove bubbles formed during the mixing of the components. Then, PDMS 20:1 was poured onto a 500 µm thick PMMA layer to facilitate handling of the thin PDMS layer. The mixture was spin-coated using a two-step program: first at 500 rpm for 5 s with a 100 rpm/s acceleration, followed by 1,400 rpm for 1 min with the same acceleration. These parameters were adjusted to achieve a final PDMS thickness of 40 µm. The spun PDMS was then baked at 80 °C for 2 h to accomplish the final crosslinking of the material. The membrane was bonded to the PMMA microfluidic circuitry using a PDMS-PMMA bonding technique adapted from a literature method (Norouzi et al., 2018). In more details, an intermediate SiO_2_ layer was created at the PMMA-PDMS interface to improve adhesion between the two parts. A chloroform-based solution was prepared by mixing 60% ethanol, 20% tetraethyl orthosilicate (TEOS, 99.999% trace metals basis, Sigma-Aldrich), 10% 0.1 M HCl and 10% chloroform. The solution was spin-coated onto the cleaned PMMA surface at 2000 rpm for 1 min with a 500 rpm/s acceleration. The SiO_2_ layer was activated on both surfaces of the PMMA and PDMS membrane using oxygen plasma treatment (Plasma Etch, Thermoservice) carried out for 5 s at a power of 100 W and flux of 15 sccm. The two treated sides were then brought into direct contact and baked in oven at 80 °C for 2 h. The PMMA-bonded PDMS was then peeled off from the 500 µm PMMA layer, following an adapted procedure from the literature (Criscuolo et al., 2020). The free surface of the PDMS was bonded to the remaining PMMA microfluidic layers to seal the platform, following the same procedure described above. The device was completed by attaching Nanoport connectors (NanoPort Kit for 1/16″ OD tubing, Darwin Microfluidics) to the micro-channels of the platform, for the culture medium and air flow.

### 5.2 Fabrication of the patterned PDMS membrane and integration in the microfluidic platform

AutoCAD modeling was used to design the radial topography consisting of ridges and grooves arranged in a circular pattern. The micropattern was fabricated using photolithographic techniques. A schematic workflow chart is reported in Supplementary Figure S11a. First, a positive photoresist, AZ 1505 (MicroChemicals GmbH), was poured and spin-coated onto the surface of a 500 µm thick PMMA layer. The photoresist was spun in a two-step process to reach a thickness of 1 μm, using 500 rpm for 10 s at 100 rpm/s acceleration in the first step and 1,000 rpm for 40 s at 1,000 rpm/s acceleration in the second step. Immediately after spin-coating, the photoresist was soft-baked at 100 °C for 50 s and exposed to a high-resolution direct-write laser lithography tool (DWL 66 fs, Heidelberg Instruments) according to the model design. After exposure, the sample was pre-baked at 100 °C for 1 min, developed using AZ 351B (MicroChemicals GmbH) for 20 s. A hard-bake at 90 °C for 50 s completed the process. After the micropattern master fabrication, PDMS 20:1 was poured and spun on the surface of the micropatterned photoresist on the 500 µm-flexible PMMA layer, using the same PDMS membrane spinning parameters. The PDMS was then cured at 80 °C for 2 h. Once cooled to room temperature, the spun PDMS was bonded to the PMMA microfluidic circuitry following the previously described PDMS-PMMA SiO_2_ treatment. Following the PDMS-PMMA bonding, the patterned PDMS, now fixed onto the microfluidic circuitry, was peeled off from the thin PMMA carrying the patterned photoresist layer. The entire device was sealed with the upper part, which contained the microfluidic channels and chambers for cell seeding. Finally, Nanoport connectors were attached, as previously described.

### 5.3 Pressure-controlled deformation

Both the unpatterned and micropatterned PDMS membranes, embedded in the microfluidic device, were mechanically stimulated by applying air suction flow. Nanoport connectors linked the platform’s microchannels to an external pressure controller via PTFE tubes. The controller (OB1 MK4, Elveflow) included internal vacuum channels remotely controlled by software, enabling the imposition of different pressure curves for the air flow with limit pressure values of ±1 bar. The system applied static deformation to the PDMS membrane by imposing a linear pressure curve with slope of 20 mbar/s. A set of negative pressure values, ranging from 25 to 300 mbar, was tested.

### 5.4 Device performance testing and data analysis

For device testing purposes, the PDMS membrane was loaded with fluorescent nanoparticles. Specifically, the uncured PDMS was mixed with fluorescent polystyrene nanobeads (Fluoro-Max Dyed Green Aqueous Fluorescent Particles, 1% solids, diameter of 0.083 µm, Thermo Scientific) at a concentration of 15 μL/g of PDMS. The PDMS was then spin-coated and cured as previously described. After integrating the membrane into the device, equibiaxial deformation was applied to the fluorescence-labeled PDMS using the pressure controller, providing continuous negative pressures over time. Fluorescence was tracked by acquiring its vertical displacement within the mechanical deformation unit chamber, capturing z-stack images with a Leica TCS SP 5 (Leica Microsystems) and using a 10x objective. The sample was excited with a 488 nm laser and emission was collected in the (495–515) nm range. In addition, time-lapse images were taken over time by tracking the positions and displacements of the fluorescent nanobeads in the x-y plane during the equibiaxial deformation in the microfluidic platform. Confocal images were also acquired, using the same parameters, while flowing an aqueous solution of fluorescent nanobeads (1:1000 nanobeads in deionized water) through the microfluidic channels at a flow rate of 100 μL/min. This approach was implemented to assess the platform’s fluid dynamic response and the interaction of fluid flow with mechanical deformation.

Confocal images from device testing were reconstructed and analyzed using ImageJ (Fiji). Z-stacks were reconstructed and the Analyze Line Graph function in ImageJ was used to retrieve the coordinates of the fluorescent-labeled PDMS membrane lowering during mechanical stimulation. These coordinates were uploaded and plotted in MATLAB. By retrieving the minimum coordinates reached by the membrane during the vertical lowering as a function of the suction pressure, calibration curves were generated.

Starting from confocal time-series of PDMS-embedded fluorescent nanobeads undergoing the equibiaxial stimulation, their trajectories were tracked using a Fiji tracking plugin, MTrackJ. A semi-automatic detection combined with manual tracing of the moving beads was performed. The detection algorithm calculated the position of a specified image feature, the bright centroid, allowing precise mouse cursor positioning. The positions of the tracked nanobeads were used to reconstruct the deformation field and map it in MATLAB.

### 5.5 PDMS membrane characterization

The mechanical response of the PDMS 20:1 was characterized to determine the elastic modulus of the material under stretching. Uniaxial traction tests were performed using an Instron testing machine on a dog-bone shaped PDMS 20:1 specimen (Supplementary Figures S9a,b). The traction experiment started with a preload of 0.1 N and a solicitation speed of 1 mm/min, and load-displacement curves were recorded. Engineering stress and strain curves were plotted based on measurements of the specimen’s cross-sectional area and gauge length. Fitting the linear region of these curves provided the elastic modulus of the material (Supplementary Figures S9a,c).

Profilometer measurements of the PDMS membrane thickness were conducted using a stylus profilometer (Dektak, Bruker) with a stylus radius of 2.5 µm and a force of 1 mg. For the PDMS membrane profiling, a scanning length of 3 mm with a duration of 60 s was used and a valley profile was measured in a range of 65.5 µm (Supplementary Figure S10a).

### 5.6 Micropatterned master and mold characterization

The photoresist master of the radial micropattern was scanned using a JPK NanoWizard II (JPK Instruments) atomic force microscope (AFM) mounted on an Axio Observer Z1 microscope (Zeiss) for cantilever positioning and scanning of the micrometric patterned area. Silicon nitride probes with a triangular tip (MLCT, Bruker) and a spring constant of 0.1 N/m were used for the scans. JPK data processing software was used to correct the raw scanning images from noise and artifacts.

Scanning electron microscopy (SEM) imaging of the patterned PDMS mold was conducted with a Zeiss Ultraplus field emission gun (FE-SEM). Imaging parameters included 5 kV high tension and 30 μm aperture. Prior to high vacuum imaging, the PDMS samples were mounted onto an aluminum stub (Electron Microscopy Sciences) by using carbon tape (Agar Scientific) and coated with a 10 nm conductive gold layer via sputtering by using HR208 sputter coater (Cressington).

For the profile imaging of the micropatterned PDMS, 1 μm cryo-slices were prepared at −120 °C using an ultramicrotome (Leica FC7-UC7) and the imaging was performed by using Helios CX5, Focused Ion Beam Scanning Electron Microscope of ThermoFisher. The slices were dried at room temperature, then mounted on aluminum stub with carbon tape and sputtered with a 10 nm coating layer of gold for SEM imaging. Also, 1 mm × 1 mm x 10 μm pieces of PDMS sample were cryo-sectioned before profile imaging of the micropattern. The pieces were mounted pre-tilted onto 12 mm aluminum stub and sputtered with a layer of 10 nm of gold. The stub was tilted again in the chamber of microscope to reach 90° tilt. The imaging of the cryo-slices and pieces was performed with 3 KV of high tension with 0.17 nA of current. The TLD detector was used in secondary electron mode in immersion mode with a magnification of 4 KX-10 KX.

### 5.7 COMSOL multiphysics modelling and simulations

COMSOL Multiphysics (version 5.0) was used to simulate the fluid dynamics and mechanical behavior of the designed microfluidic platform. 3D simulations of the Fluid Flow physics were performed to model the culture medium flow within a high aspect ratio chamber. The Laminar Flow interface was used to solve the fluid dynamics equations, with the culture medium approximated as water for simplicity. Additional details on the simulation parameters are provided in Supplementary Table S1. The working principle of the mechanical stimulation unit was simulated by using multiphysics coupling to combine air flow and flexible membrane deformations. The air flow was modeled with the Laminar Flow interface, assuming air as a compressible fluid with Ma<0.3. Additional assumptions and parameters for air flow physics can be found in Supplementary Table S2 and Supplementary Figure S2b. Membrane deformation was modelled using the Structural Mechanics physics through the Solid Mechanics interface, with PDMS as the material. The material behavior was approximated as hyperelastic, following a Neo-Hookean model. Characteristic parameters of the material such as density and Poisson’s ratio were obtained from previous literature studies (Ho et al., 2018; Briones et al., 2021; Zhang et al., 2022), while the Young’s Modulus was based on the results of the uniaxial traction test conducted on the PDMS 20:1. The same approach was used for the Neo-Hookean model parameters, i.e., Bulk modulus and Lamé parameter (Kim et al., 2011; Briones et al., 2021; Zhang et al., 2022). The values used for the structural mechanics simulations, along with model assumptions, are provided in Supplementary Table S3. In the structural analysis, the load applied to the bottom and top sides of the PDMS membrane was the pressure exerted by the suction air flow in the underlying deformation chamber and the static air flow on the top, respectively. Differently, in the coupling of fluid flow and mechanical stimulation, water flow and Laminar flow interface were used for the upper microfluidic chambers, and the upper load on the PDMS membrane was set as the pressure exerted by the upper flowing water (Supplementary Figure S7). Fixed constraints were applied for membrane movement during the simulation, including null displacements on the top of the pillar surface and on the side of the membrane (Supplementary Figures S2c, S7d). A physics-controlled finer mesh was used for all the simulations.

### 5.8 HL-1 cell culture

HL-1 CMs were seeded following the protocols outlined by Claycomb et al. (1998) Cells were seeded and left to grow in T-25 flask with Claycomb medium (51800C - Merck), supplemented with 10% fetal bovine serum (FBS) (F7524 - Merck), 1% Pen/Strep at a final concentration of 100 units of penicillin/mL and 100 µg streptomycin/mL (L0022 - Microtech), 1% L-glutamine 200 mM (G7513 – Merck) at a final concentration of 2 mM, and 1% norepinephrine solution at a final concentration of 0.1 mM. The norepinephrine solution was used to maintain the contractility phenotype of the HL-1 CMs and was prepared by dissolving 80 mg of norepinephrine powder ((+/−)-norepinephrine (+)-bitartrate salt, A0937 – Merck) in 20 mL of a 30 mM ascorbic acid aqueous solution. After medium preparation, it was stored in the dark and used to feed the cells every 2 days while letting them grow in the incubator set at 37 °C, 5% CO_2_ and 95% humidity.

### 5.9 HL-1 cells seeding in the microfluidic platform

The microfluidic device was sterilized by flushing various solutions through the inlet and outlet microchannels, as well as the cell seeding chamber, to ensure sterility in the areas where the culture medium flows. First, 70% ethanol was flushed and left for 5 min, followed by three washes with deionized water. Next, a solution of the antibiotic Pen/Strep in PBS at a 1:2 ratio was used to rinse the channels and chambers, followed by washes with PBS. The device and its microchannels were left to dry under laminar flow in a biological hood under sterile conditions and were further exposed to UV light for 1 h to sterilize the external surfaces of the platform. After the complete evaporation of the solvents, the PDMS embedded in the device was functionalized with fibronectin, just before cell seeding. A solution of 0.1% fibronectin from human plasma (F0895 – Merck) in sterile PBS was prepared and flushed into the micro-channels. The device was incubated at 37 °C for 1 h, allowing the fibronectin protein to precipitate and coat the surface of the PDMS. After the incubation time, the fibronectin solution was removed, and the channels and chambers were washed twice with complete cell culture medium. Then, complete culture medium was left in the device, which was incubated at 37 °C until the cell seeding procedure, enabling other proteins from the culture medium to adsorb on the surface of the functionalized PDMS.

Once HL-1 cells grew and detached from T-25 flask, they were counted and seeded into the sterile microfluidic device at a seeding density of 2⋅10^5^ cells/mL. The cell suspension was introduced into the inlet channel of the device to completely fill the seeding chamber, as well as the inlet and outlet channels. The device was incubated at 37 °C, 5% CO_2_ and 95% humidity, allowing the cells to adhere to the functionalized PDMS. Afterward, the medium exchange was performed daily with pre-warmed complete culture medium by continuous flow at 10 μL/min.

### 5.10 HL-1 cells stimulation

For experiments related to topography alignment quantification, HL-1 cells were seeded on micropatterned PDMS within the device. Morphological alignment was assessed 24 h after seeding by fixing the samples and staining f-actin and nuclei. In migration experiments, HL-1 cells were seeded on devices containing both flat and patterned PDMS. After 24 h of cell seeding, the devices were moved to a microscope-associated incubation chamber with controlled temperature, maintained at 37 °C during the stimulation experiments. To maintain pH conditions suitable for cell culture, a 1M Hepes solution was added to the complete culture medium and a continuous flow at 10 μL/min was introduced during the stimulation experiment to guarantee constant nutrients and gas exchange. The OB1 MK4 pressure controlled was placed near the incubation chamber of the confocal microscope to apply the stimulation during the acquisition process. In particular, The air flow channel was connected to the OB1 external pressure controller, and a continuous pressure of −300 mbar was applied to the device for 1 h to deform the PDMS substrate and apply equibiaxial stimulation to the HL-1 cells seeded on top of the pillar area. Nuclei were stained and tracked.

### 5.11 HL-1 cells staining and imaging

Cells were stained and tracked with confocal microscopy. For assessing the response of HL-1 cells to fibronectin functionalization, cells were stained with Hoechst 33342 (H3570 – Invitrogen) in PBS (1:10,000) for 10 min. Images were acquired using a ×10 objective on an EVOS microscope (FL Cell Imaging System, ThermoFisher Scientific) during live imaging sessions. This procedure was repeated at 24, 48 and 72 h after substrate functionalization and cell seeding. The same Hoechst 33342 staining procedure was used to track the migration of HL-1 cells on flat and patterned device-embedded PDMS under mechanical deformation for 1 h. Live-imaging of cell nuclei was conducted using a confocal Laser Scanning Microscope (LSM) 900 model (Zeiss), with a 10x objective. Hoechst was excited with a 350 nm laser, and brightfield images of the PDMS membrane on the device pillar were acquired to track the precise positions of cells during the migration.

For live-dead assay, HL-1 cells were stained to detect vital and non-vital cells in response to topographic signal, using Calcein AM (final concentration 1 μg/mL) and Propidium Iodide (PI, final concentration 10 μg/mL), simultaneously. Samples were washed incubated for 15 min in the solution containing both the probes in PBS. Then, the samples were rinsed in culture medium for live-imaging acquisition, performed with a Leica TCS SP 5 (Leica Microsystems). Calcein AM was excited with a 488 nm laser, emitting in the range (495–515) nm, while PI was excited at 555 nm and read in the range of emission (528–617) nm. Both fluorescence and brightfield images were captured to detect the micropattern, all with a 10x objective.

For alignment experiments on patterned PDMS, cells were seeded for 24 h and then fixed, following the procedure outlined below. Cells were fixed in 4% paraformaldehyde (PFA) in PBS for 15 min at room temperature. PFA was removed and the samples were washed in PBS. 0.1% Triton X-100 in PBS was used for 10 min at 4 °C. Samples were washed twice in PBS and stained with a solution of Hoechst 33342 in PBS 1:10,000 (20 min) and a solution of Phalloidin 555 (Abcam) in PBS at 1:1000 (1 h) in a light-shielded environment, after which the samples were washed and preserved in PBS under humid conditions at 4 °C. Imaging of the samples was carried out through a confocal Laser Scanning Microscope (LSM) 900 model (Zeiss) with a 10x objective. Phalloidin was excited with a 555 nm laser and acquired in the range (570–600) nm, while Hoechst was excited at 350 nm and acquired up to 461 nm. Brightfield images were also acquired to define the precise position and orientation of the cells relative to the micropattern.

### 5.12 HL-1 cells sample preparation and imaging by focused ion beam scanning electron microscopy

Flat and micropatterned PDMS substrates seeded for 24 h with HL-1 cells were prepared for FIB-SEM imaging according to the ROTO protocol and an ultra-thin plasticization protocol (Li et al., 2019). Samples were fixed in 2.5% glutaraldehyde in 0.1 M sodium cacodylate buffer at 4 °C overnight, then they were washed three times in 0.1 M sodium cacodylate. After quenching in 20 mM glycine for 20 min at 4 °C and washing in sodium cacodylate, samples were incubated in 1% osmium tetroxide/1% potassium ferrocyanide for 1 h at 4 °C in the dark, washed in sodium cacodylate and incubated in 1% thiocarbohydrazide aqueous solution for 20 min at room temperature in the dark. Specimens were washed three times in distilled water before incubation with 1% osmium tetroxide aqueous solution and they were incubated in a 1% uranyl acetate solution overnight, in the dark, at 4 °C. Before dehydration samples were washed in distilled water, incubated in 0.15% tannic acid solution, then washed three times in chilled water. The dehydration was performed by increasing concentration of ethanol (30%, 50%, 70%, 90%, 100%), each step of the duration of 15 min on ice followed by three steps in absolute ethanol at room temperature. Specimen were infiltrated in low viscosity resin and absolute ethanol using this ratio 1:3 (two times for 3 h), 1:2 (two times for 3 h), 1:1 (overnight), 1:2 (two times for 3 h), 2:1 (two times for 3 h), 3:1 (two times for 3 h) before infiltration in absolute resin for one night and 1 day. The excess of resin was removed by washing with ethanol before polymerization in oven at 70 °C for 30 h. Samples were mounted on 12 mm aluminum stub by using silver paste then sputtered with 20 nm of gold. The imaging was performed by using Helios CX 5 (ThemoFisher) at 3 KV in a range of magnification between 5 KX and 50KX. The cross sections were obtained by milling with ion beam at 30 kV with a range of current of 0.23 ÷ 0.43 nA, and images were collected in immersion mode, with TLD detector in backscattered mode.

### 5.13 Image and data analysis

All confocal images were processed and analyzed using ImageJ, and quantification plots were generated with MATLAB codes. Curves of cell proliferation over time were built by automatically counting nuclei after image thresholding, using the ImageJ Analyze particles command. Percentage values of cell proliferation were calculated by normalizing the cell count to the number of seeded cells at time zero.

Live-dead quantification was performed using Ilastik, an open-source software for image segmentation and classification. The pixel classification tool was used to assign labels to the pixel of the image based on some features. Training the classifier involved iteratively marking objects to be recognized, assigning individual labels, i.e., pixel classes, for live cells (green color), dead cells (red color), and background (black). After running feature selection and extracting the automatic segmentation, live and dead cells were separated into distinct images and counted using the Analyze particles tool of ImageJ. The values were normalized against the total cell count, considering both live and dead cells.

The Directionality plugin of ImageJ was used to extract the dominant orientation of nuclei and f-actin from individual images. By extracting the direction values and by plotting their histogram in MATLAB, a fitting with a Gaussian distribution was performed to assess the mean angular alignment. This alignment reflects the difference between the mean orientation of nucleus or f-actin in a single sector and the sector’s orientation. A linear fit was also applied to the orientation of nuclei and f-actin with respect to the sector orientation. From f-actin images, the call spreading area and aspect ratio were evaluated for HL-1 cells, seeded on both flat and patterned substrates without and with equibiaxial mechanical deformation. Thresholding and then ellipse fitting were used to first quantify the area with the cell boundaries and then extract minor and major cell axes for aspect ratio evaluation.

TrackMate, an ImageJ tracking tool, was used to automate the segmentation and tracking of HL-1 cell migration on the deformed PDMS under equibiaxial mechanical stimulation. A Laplacian of Gaussian (LoG) detector was employed for the cell detection, by recognizing local maxima in the filtered image. The detected spots were then linked using a two-steps algorithm, the first for trajectory segment creation and the second for closing any potential gaps between consecutive segments. After completing the tracking and extracting trajectory positions over time, the Chemotaxis and Migration tool in ImageJ was used to build Windrose diagrams. Mean cell velocities were retrieved from the positions and plotted as lognormal probability density functions in MATLAB. The peak values of the probability distributions represented the mean velocities for each group of migrating cells. Based on the track positions, turning angles of migrating cells were computed as the differences between two consecutive polar angles and were plotted as histograms in MATLAB.

### 5.14 Statistical data analysis

The experiments were performed three times per group, on three different microfluidic devices, and the results were statistically tested by using a one-way ANOVA in MATLAB to find out significant differences within each group. Statistical significance was set at p < 0.05, with values below this threshold considered significant. Mean values are represented as mean ± SEM. For the turning angles distribution analysis, a non-parametric, multimodal Kuiper’s test was used to compare the distributions, as these angular data fall in the circular statistics assumption. P values were calculated between pairwise comparisons performed with the Holm-Bonferroni method as correction. Kruskal-Wallis non-parametric statistical analysis followed by post-hoc multiple comparisons was performed to evaluate differences in cell area and aspect ratio for cells seeded on flat and patterned substrate, with and without deformation, for 1h, 2h and 3 h.

## Data Availability

The raw data supporting the conclusions of this article will be made available by the authors, without undue reservation.

## References

[B1] Ahn H. Cho Y. Yun G. T. Jung K. B. Jeong W. Kim Y. (2023). Hierarchical topography with tunable Micro- and nanoarchitectonics for highly enhanced cardiomyocyte maturation via multi-scale mechanotransduction. Adv. Healthc. Mater 12, 2202371. 10.1002/ADHM.202202371 36652539

[B2] Akther F. Yakob S. B. Nguyen N. T. Ta H. T. (2020). Surface modification techniques for endothelial cell seeding in PDMS microfluidic devices. Biosens. (Basel) 10, 182. 10.3390/BIOS10110182 33228050 PMC7699314

[B3] Bamshad A. Nikfarjam A. Khaleghi H. (2016). A new simple and fast thermally-solvent assisted method to bond PMMA–PMMA in micro-fluidics devices. J. Micromechanics Microengineering 26, 065017. 10.1088/0960-1317/26/6/065017

[B4] Bijnens B. Cikes M. Butakoff C. Sitges M. Crispi F. (2012). Myocardial motion and deformation: what does it tell us and how does it relate to function? Fetal Diagn Ther. 32, 5–16. 10.1159/000335649 22584107

[B5] Boycott H. E. Barbier C. S. M. Eichel C. A. Costa K. D. Martins R. P. Louault F. (2013). Shear stress triggers insertion of voltage-gated potassium channels from intracellular compartments in atrial myocytes. Proc. Natl. Acad. Sci. U. S. A. 110, E3955–E3964. 10.1073/pnas.1309896110 24065831 PMC3799377

[B6] Briones J. Espulgar W. Koyama S. Takamatsu H. Tamiya E. Saito M. (2021). A design and optimization of a high throughput valve based microfluidic device for single cell compartmentalization and analysis. Sci. Rep. 2021 11, 12995–12. 10.1038/s41598-021-92472-w 34155296 PMC8217553

[B7] Buskermolen A. B. C. Ristori T. Mostert D. van Turnhout M. C. Shishvan S. S. Loerakker S. (2020). Cellular contact guidance emerges from gap avoidance. Cell Rep. Phys. Sci. 1, 100055–20. 10.1016/j.xcrp.2020.100055 32685934 PMC7357833

[B8] Butcher J. T. Barrett B. C. Nerem R. M. (2006). Equibiaxial strain stimulates fibroblastic phenotype shift in smooth muscle cells in an engineered tissue model of the aortic wall. Biomaterials 27, 5252–5258. 10.1016/J.BIOMATERIALS.2006.05.040 16806457

[B9] Chagnon-Lessard S. Jean-Ruel H. Godin M. Pelling A. E. (2017). Cellular orientation is guided by strain gradients. Integr. Biol. 9, 607–618. 10.1039/C7IB00019G 28534911

[B10] Chelnokova N. O. Golyadkina A. A. Kirillova I. V. Polienko A. V. Ivanov D. V. (2016). Morphology and biomechanics of human heart, 9710, 971013–971132. 10.1117/12.2208423

[B11] Choi S. Hong Y. Lee I. Huh D. Jeon T. J. Kim S. M. (2013). Effects of various extracellular matrix proteins on the growth of HL-1 cardiomyocytes. Cells Tissues Organs 198, 349–356. 10.1159/000358755 24662367

[B12] Claycomb W. C. Lanson N. A. Stallworth B. S. Egeland D. B. Delcarpio J. B. Bahinski A. (1998). HL-1 cells: a cardiac muscle cell line that contracts and retains phenotypic characteristics of the adult cardiomyocyte. Proc. Natl. Acad. Sci. U. S. A. 95, 2979–2984. 10.1073/PNAS.95.6.2979 9501201 PMC19680

[B13] Criscuolo V. Montoya N. A. Lo Presti A. Occhipinti L. G. Netti P. A. Vecchione R. (2020). Double-framed thin elastomer devices. ACS Appl. Mater Interfaces 12, 55255–55261. 10.1021/acsami.0c16312 33252224 PMC7735669

[B14] Dalby M. J. Gadegaard N. Oreffo R. O. C. (2014). Harnessing nanotopography and integrin–matrix interactions to influence stem cell fate. Nat. Mater. 13, 558–569. 10.1038/nmat3980 24845995

[B15] Dwyer K. D. Coulombe K. L. K. (2021). Cardiac mechanostructure: using mechanics and anisotropy as inspiration for developing epicardial therapies in treating myocardial infarction. Bioact. Mater 6, 2198–2220. 10.1016/J.BIOACTMAT.2020.12.015 33553810 PMC7822956

[B16] Eschweiler J. Horn N. Rath B. Betsch M. Baroncini A. Tingart M. (2021). The biomechanics of cartilage—an overview. Life 11, 302. 10.3390/LIFE11040302 33915881 PMC8065530

[B17] Espina J. A. Cordeiro M. H. Milivojevic M. Pajić-Lijaković I. Barriga E. H. (2023). Response of cells and tissues to shear stress. J. Cell Sci. 136, jcs260985. 10.1242/JCS.260985 37747423 PMC10560560

[B18] Faghih M. M. Sharp M. K. (2018). Solvent-based bonding of PMMA–PMMA for microfluidic applications. Microsyst. Technol. 25, 3547–3558. 10.1007/S00542-018-4266-7

[B19] Friedrich O. Merten A. L. Schneidereit D. Guo Y. Schürmann S. Martinac B. (2019). Stretch in focus: 2D inplane cell stretch systems for studies of cardiac mechano-signaling. Front. Bioeng. Biotechnol. 7, 55. 10.3389/fbioe.2019.00055 30972334 PMC6445849

[B20] Gardel M. L. Schneider I. C. Aratyn-Schaus Y. Waterman C. M. (2010). Mechanical integration of actin and adhesion dynamics in cell migration. Annu. Rev. Cell Dev. Biol. 26, 315–333. 10.1146/ANNUREV.CELLBIO.011209.122036 19575647 PMC4437624

[B21] Ge L. Yang L. Bron R. Burgess J. K. Van Rijn P. (2020). Topography-mediated fibroblast cell migration is influenced by direction, wavelength, and amplitude. ACS Appl. Bio Mater. 3 (4), 2104–2116. 10.1021/acsabm.0c00001 35025262

[B22] Gonnerman E. A. Kelkhoff D. O. McGregor L. M. Harley B. A. C. (2012). The promotion of HL-1 cardiomyocyte beating using anisotropic collagen-GAG scaffolds. Biomaterials 33, 8812–8821. 10.1016/J.BIOMATERIALS.2012.08.051 22979989

[B23] Gould R. A. Chin K. Santisakultarm T. P. Dropkin A. Richards J. M. Schaffer C. B. (2012). Cyclic strain anisotropy regulates valvular interstitial cell phenotype and tissue remodeling in three-dimensional culture. Acta Biomater. 8, 1710–1719. 10.1016/J.ACTBIO.2012.01.006 22281945 PMC3678539

[B24] Guetta-Terrier C. Monzo P. Zhu J. Long H. Venkatraman L. Zhou Y. (2015). Protrusive waves guide 3D cell migration along nanofibers. J. Cell Biol. 211 (3), 683–701. 10.1083/jcb.201501106 26553933 PMC4639865

[B25] Guvendiren M. Burdick J. A. (2013). Stem cell response to spatially and temporally displayed and reversible surface topography. Adv. Healthc. Mater 2, 155–164. 10.1002/ADHM.201200105 23184470

[B26] Han S.-B. Kim J.-K. Lee G. Kim D.-H. Han S.-B. Kim J.-K. (2020). Mechanical properties of materials for stem cell differentiation. Adv. Biosyst. 4, 2000247. 10.1002/ADBI.202000247 33035411

[B27] Ho K. K. Y. Wang Y. L. Wu J. Liu A. P. (2018). Advanced microfluidic device designed for cyclic compression of single adherent cells. Front. Bioeng. Biotechnol. 6, 148. 10.3389/fbioe.2018.00148 30386779 PMC6198036

[B28] Hu X. Liu H. Li M. Zhu J. Yu Z. (2021). Transcriptomic analysis reveals the role of a peptide derived from CRYAB on the CoCl2-induced hypoxic HL-1 cardiomyocytes. J. Thromb. Thrombolysis 51, 265–276. 10.1007/s11239-020-02117-4 32621152

[B29] Huang G. Li F. Zhao X. Ma Y. Li Y. Lin M. (2017). Functional and biomimetic materials for engineering of the three-dimensional cell microenvironment. Chem. Rev. 117, 12764–12850. 10.1021/acs.chemrev.7b00094 28991456 PMC6494624

[B30] Ingber D. E. (2022). Human organs-on-chips for disease modelling, drug development and personalized medicine. Nat. Rev. Genet. 23, 467–491. 10.1038/s41576-022-00466-9 35338360 PMC8951665

[B31] Jafari A. Behjat E. Malektaj H. Mobini F. (2023). Alignment behavior of nerve, vascular, muscle, and intestine cells in two- and three-dimensional strategies. WIREs Mech. Dis. 15, e1620. 10.1002/WSBM.1620 37392045

[B32] Kim T. K. Kim J. K. Jeong O. C. (2011). Measurement of nonlinear mechanical properties of PDMS elastomer. Microelectron. Eng. 88, 1982–1985. 10.1016/J.MEE.2010.12.108

[B33] Kim J. Shanmugasundaram A. Lee C. B. Kim J. R. Park J. J. Kim E. S. (2023). Enhanced cardiomyocyte structural and functional anisotropy through synergetic combination of topographical, conductive, and mechanical stimulation. Lab. Chip 23, 4540–4551. 10.1039/D3LC00451A 37771289

[B34] Krishnan R. Canović E. P. Iordan A. L. Rajendran K. Manomohan G. Pirentis A. P. (2012). Fluidization, resolidification, and reorientation of the endothelial cell in response to slow tidal stretches. Am. J. Physiol. Cell Physiol. 303 (4), C368–C375. 10.1152/ajpcell.00074.2012 22700796 PMC3422985

[B35] Kumar A. Chaudhry I. Reid M. B. Boriek A. M. (2002). Distinct signaling pathways are activated in response to mechanical stress applied axially and transversely to skeletal muscle fibers. J. Biol. Chem. 277, 46493–46503. 10.1074/JBC.M203654200 12221078

[B36] Lawless B. M. Sadeghi H. Temple D. K. Dhaliwal H. Espino D. M. Hukins D. W. L. (2017). Viscoelasticity of articular cartilage: analysing the effect of induced stress and the restraint of bone in a dynamic environment. J. Mech. Behav. Biomed. Mater 75, 293–301. 10.1016/J.JMBBM.2017.07.040 28763685 PMC5636614

[B37] Leung C. M. de Haan P. Ronaldson-Bouchard K. Kim G. A. Ko J. Rho H. S. (2022). A guide to the organ-on-a-chip. Nat. Rev. Methods Prim. 2022 2 (1), 33–29. 10.1038/s43586-022-00118-6

[B38] Li X. Matino L. Zhang W. Klausen L. McGuire A. F. Lubrano C. (2019). A nanostructure platform for live-cell manipulation of membrane curvature. Nat. Protoc. 14 (6), 1772–1802. 10.1038/s41596-019-0161-7 31101905 PMC6716504

[B39] Liao H. Qi Y. Ye Y. Yue P. Zhang D. Li Y. (2021). Mechanotranduction pathways in the regulation of mitochondrial homeostasis in cardiomyocytes. Front. Cell Dev. Biol. 8, 625089. 10.3389/fcell.2020.625089 33553165 PMC7858659

[B40] Marchioni A. Tonelli R. Cerri S. Castaniere I. Andrisani D. Gozzi F. (2021). Pulmonary stretch and lung mechanotransduction: implications for progression in the fibrotic lung. Int. J. Mol. Sci. 22, 6443–22. 10.3390/IJMS22126443 34208586 PMC8234308

[B41] Marsano A. Conficconi C. Lemme M. Occhetta P. Gaudiello E. Votta E. (2016). Beating heart on a chip: a novel microfluidic platform to generate functional 3D cardiac microtissues. Lab. Chip 16, 599–610. 10.1039/C5LC01356A 26758922

[B42] Mazzio E. A. Soliman K. F. A. (2012). Basic concepts of epigenetics impact of environmental signals on gene expression. Epigenetics 7, 119–130. 10.4161/epi.7.2.18764 22395460 PMC3335905

[B43] Melo-Fonseca F. Carvalho O. Gasik M. Miranda G. Silva F. S. (2023). Mechanical stimulation devices for mechanobiology studies: a market, literature, and patents review. Biodes Manuf. 6, 340–371. 10.1007/s42242-023-00232-8

[B44] Merino-Casallo F. Gomez-Benito M. J. Hervas-Raluy S. Garcia-Aznar J. M. (2022). Unravelling cell migration: defining movement from the cell surface. Cell Adh Migr. 16, 25–64. 10.1080/19336918.2022.2055520 35499121 PMC9067518

[B45] Miranda I. Souza A. Sousa P. Ribeiro J. Castanheira E. M. S. Lima R. (2021). Properties and applications of PDMS for biomedical engineering: a review. J. Funct. Biomater. 13, 2. 10.3390/JFB13010002 35076525 PMC8788510

[B46] Molnar K. Labouesse M. (2021). The plastic cell: mechanical deformation of cells and tissues. Open Biol. 11, 210006. 10.1098/RSOB.210006/ 33529554 PMC8061695

[B47] Moraes C. Chen J. H. Sun Y. Simmons C. A. (2009). Microfabricated arrays for high-throughput screening of cellular response to cyclic substrate deformation. Lab. Chip 10, 227–234. 10.1039/B914460A 20066251

[B48] Nam K. H. Kim P. Wood D. K. Kwon S. Provenzano P. P. Kim D. H. (2016). Multiscale cues drive collective cell migration. Sci. Rep. 2016 6 (1), 29749–13. 10.1038/srep29749 27460294 PMC4962098

[B49] Navaee F. Renaud P. Piacentini N. Durand M. Bayat D. Z. Ledroit D. (2023). Toward a physiologically relevant 3D helicoidal-oriented cardiac model: simultaneous application of mechanical stimulation and surface topography. Bioengineering 10, 266. 10.3390/bioengineering10020266 36829760 PMC9952807

[B50] Nguyen A. T. Sathe S. R. Yim E. K. F. (2016). From nano to micro: topographical scale and its impact on cell adhesion, morphology and contact guidance. J. Phys. Condens. Matter 28, 183001. 10.1088/0953-8984/28/18/183001 27066850

[B51] Nitsan I. Drori S. Lewis Y. E. Cohen S. Tzlil S. (2016). Mechanical communication in cardiac cell synchronized beating. Nat. Phys. 12 (5), 472–477. 10.1038/nphys3619

[B52] Norouzi A. R. Nikfarjam A. Hajghassem H. (2018). PDMS–PMMA bonding improvement using SiO2 intermediate layer and its application in fabricating gas micro valves. Microsyst. Technol. 24, 2727–2736. 10.1007/s00542-017-3676-2

[B53] Parker K. K. Ingber D. E. (2007). Extracellular matrix, mechanotransduction and structural hierarchies in heart tissue engineering. Philos. Trans. R. Soc. Lond B Biol. Sci. 362, 1267–1279. 10.1098/RSTB.2007.2114 17588874 PMC2440395

[B54] Raftopoulou M. Hall A. (2004). Cell migration: rho GTPases lead the way. Dev. Biol. 265, 23–32. 10.1016/j.ydbio.2003.06.003 14697350

[B55] Riching K. M. Cox B. L. Salick M. R. Pehlke C. Riching A. S. Ponik S. M. (2015). 3D collagen alignment limits protrusions to enhance breast cancer cell persistence. Biophys. J. 107, 2546–2558. 10.1016/j.bpj.2014.10.035 25468334 PMC4255204

[B56] Ronaldson-Bouchard K. Ma S. P. Yeager K. Chen T. Song L. J. Sirabella D. (2018). Advanced maturation of human cardiac tissue grown from pluripotent stem cells. Nature 556, 239–243. 10.1038/s41586-018-0016-3 29618819 PMC5895513

[B57] Rubin D. Krempl E. (2010). Introduction to continuum mechanics. Elsevier Inc. 10.1016/B978-0-7506-8560-3.X0001-1

[B58] Sackmann E. K. Fulton A. L. Beebe D. J. (2014). The present and future role of microfluidics in biomedical research. Nature 507, 181–189. 10.1038/nature13118 24622198

[B59] Schäfer C. Faust U. Kirchgeßner N. Merkel R. Hoffmann B. (2011). The filopodium: a stable structure with highly regulated repetitive cycles of elongation and persistence depending on the actin cross-linker fascin. Cell Adh Migr. 5, 431–438. 10.4161/CAM.5.5.17400 21975552 PMC3218610

[B60] Seo C. H. Jeong H. Furukawa K. S. Suzuki Y. Ushida T. (2013). The switching of focal adhesion maturation sites and actin filament activation for MSCs by topography of well-defined micropatterned surfaces. Biomaterials 34, 1764–1771. 10.1016/J.BIOMATERIALS.2012.11.031 23219606

[B61] Shao Y. Mann J. M. Chen W. Fu J. (2014). Global architecture of the F-actin cytoskeleton regulates cell shape-dependent endothelial mechanotransduction. Integr. Biol. (Camb) 6, 300. 10.1039/C3IB40223A 24435061 PMC3963173

[B62] Shellard A. Mayor R. (2020). Rules of collective migration: from the wildebeest to the neural crest. Philosophical Trans. R. Soc. B 375, 20190387. 10.1098/RSTB.2019.0387 32713298 PMC7423382

[B63] Siddique A. B. Shanmugasundaram A. Kim J. Y. Roshanzadeh A. Kim E. S. Lee B. K. (2022). The effect of topographical and mechanical stimulation on the structural and functional anisotropy of cardiomyocytes grown on a circular PDMS diaphragm. Biosens. Bioelectron. 204, 114017. 10.1016/J.BIOS.2022.114017 35158156

[B65] Slater J. H. Boyce P. J. Jancaitis M. P. Gaubert H. E. Chang A. L. Markey M. K. (2015). Modulation of endothelial cell migration via manipulation of adhesion site growth using nanopatterned surfaces. ACS Appl. Mater Interfaces 7, 4390–4400. 10.1021/AM508906F 25625303

[B66] Smiseth O. A. (2018). Evaluation of left ventricular diastolic function: state of the art after 35 years with doppler assessment. J. Echocardiogr. 16 (2), 55–64. 10.1007/s12574-017-0364-2 29236226 PMC5966482

[B67] Sommer G. Schriefl A. J. Andrä M. Sacherer M. Viertler C. Wolinski H. (2015). Biomechanical properties and microstructure of human ventricular myocardium. Acta Biomater. 24, 172–192. 10.1016/J.ACTBIO.2015.06.031 26141152

[B69] Sun Z. Costell M. Fässler R. (2019). Integrin activation by talin, kindlin and mechanical forces. Nat. Cell Biol. 21 (1), 25–31. 10.1038/s41556-018-0234-9 30602766

[B70] Tabdanov E. D. Puram V. Zhovmer A. Provenzano P. P. (2018). Microtubule-actomyosin mechanical cooperation during contact guidance sensing. Cell Rep. 25, 328–338.e5. 10.1016/j.celrep.2018.09.030 30304674 PMC6226003

[B71] Takemoto F. Uchida-Fukuhara Y. Kamioka H. Okamura H. Ikegame M. (2023). Mechanical stretching determines the orientation of osteoblast migration and cell division. Anat. Sci. Int. 98, 521–528. 10.1007/s12565-023-00716-8 37022568 PMC10366257

[B72] Tamiello C. Buskermolen A. B. C. Baaijens F. P. T. Broers J. L. V. Bouten C. V. C. (2016). Heading in the right direction: understanding cellular orientation responses to complex biophysical environments. Cell Mol. Bioeng. 9, 12–37. 10.1007/S12195-015-0422-7 26900408 PMC4746215

[B73] Teixeira A. I. McKie G. A. Foley J. D. Bertics P. J. Nealey P. F. Murphy C. J. (2006). The effect of environmental factors on the response of human corneal epithelial cells to nanoscale substrate topography. Biomaterials 27, 3945–3954. 10.1016/J.BIOMATERIALS.2006.01.044 16580065 PMC4820342

[B74] Thompson C. L. Fu S. Knight M. M. Thorpe S. D. (2020). Mechanical stimulation: a crucial element of organ-on-chip models. Front. Bioeng. Biotechnol. 8, 602646. 10.3389/fbioe.2020.602646 33363131 PMC7758201

[B75] Van Spreeuwel A. C. C. Bax N. A. M. Bastiaens A. J. Foolen J. Loerakker S. Borochin M. (2014). The influence of matrix (an)isotropy on cardiomyocyte contraction in engineered cardiac microtissues. Integr. Biol. 6, 422–429. 10.1039/C3IB40219C 24549279

[B76] Ventre M. Natale C. F. Rianna C. Netti P. A. (2014). Topographic cell instructive patterns to control cell adhesion, polarization and migration. J. R. Soc. Interface 11, 20140687. 10.1098/RSIF.2014.0687 25253035 PMC4191099

[B77] Vining K. H. Mooney D. J. (2017). Mechanical forces direct stem cell behaviour in development and regeneration. Nat. Rev. Mol. Cell Biol. 18 (12), 728–742. 10.1038/nrm.2017.108 29115301 PMC5803560

[B78] Wang J. H. C. Yang G. Li Z. Shen W. (2004). Fibroblast responses to cyclic mechanical stretching depend on cell orientation to the stretching direction. J. Biomech. 37, 573–576. 10.1016/j.jbiomech.2003.09.011 14996570

[B79] Wang D. Zheng W. Xie Y. Gong P. Zhao F. Yuan B. (2014). Tissue-specific mechanical and geometrical control of cell viability and actin cytoskeleton alignment. Sci. Rep. 4 (1), 6160–6166. 10.1038/srep06160 25146956 PMC4141254

[B80] Waters C. M. Sporn P. H. S. Liu M. Fredberg J. J. (2002). Cellular biomechanics in the lung. Am. J. Physiology-Lung Cell. Mol. Physiology 283, 503–509. 10.1152/AJPLUNG.00141.2002 12169567

[B81] Wheatley B. B. (2020). Investigating passive muscle mechanics with biaxial stretch. Front. physiology 11, 1021. 10.3389/fphys.2020.01021 32973555 PMC7468495

[B82] Wu Q. Liu J. Wang X. Feng L. Wu J. Zhu X. (2020). Organ-on-a-chip: recent breakthroughs and future prospects. Biomed. Eng. OnLine 19 (1), 9–19. 10.1186/S12938-020-0752-0 32050989 PMC7017614

[B83] Yamada K. M. Sixt M. (2019). Mechanisms of 3D cell migration. Nat. Rev. Mol. Cell Biol. 20 (12), 738–752. 10.1038/s41580-019-0172-9 31582855

[B84] Zhang D. Yang J. Hirai Y. Kamei K. I. Tabata O. Tsuchiya T. (2022). Microfabrication of polydimethylsiloxane–parylene hybrid microelectrode array integrated into a multi-organ-on-a-chip. Jpn. J. Appl. Phys. 62, 017002. 10.35848/1347-4065/ACA265

[B85] Zhao L. Sang C. Yang C. Zhuang F. (2011). Effects of stress fiber contractility on uniaxial stretch guiding mitosis orientation and stress fiber alignment. J. Biomech. 44, 2388–2394. 10.1016/J.JBIOMECH.2011.06.033 21767844

