# Supplementary material for: A multimodal microfluidic-based platform integrating topographical and equibiaxial mechanical cues for next-generation in vitro cell microenvironment mimicking
Source: Front Bioeng Biotechnol. 2025 Oct 17;13:1657107. doi: 10.3389/fbioe.2025.1657107 (PMC12575367; doi:10.3389/fbioe.2025.1657107)
Supplement: Supplementary file 1 [file DataSheet1.pdf]

Supplementary material

A multimodal microfluidic-based platform integrating topographical and equibiaxial mechanical cues for next-generation in vitro cell microenvironment mimicking

**Fluid Dynamics (spf): Water (Materials)**<br>**Laminar flow**			
**Fluid Properties**		**From material (37°C)**	
Dynamic viscosity (37°C)	μ_{water}	$6.91 \cdot 10^{-4}$	Pa·s
Density (37°C)	ρ_{water}	$9.93 \cdot 10^{2}$	kg/m^3
**Wall**		**Boundary condition: No slip**	
**Inlet**	Flow rate	Q1 = 1	µL/min
		Q2 = 10	µL/min
		Q3 = 100	µL/min
		**Boundary condition: Fully-developed flow**	
**Outlet**	Pressure	0	Pa
		**Pressure conditions: Suppress backflow**	
**Additional Parameters**	Velocity gradient	spf.sr	1/s
	Shear Stress	$\tau = \mu \cdot \mathrm{spf.sr}$	Pa·s

Table S1. Simulation parameters for the fluid dynamic operation of the platform microfluidic channels and chambers.

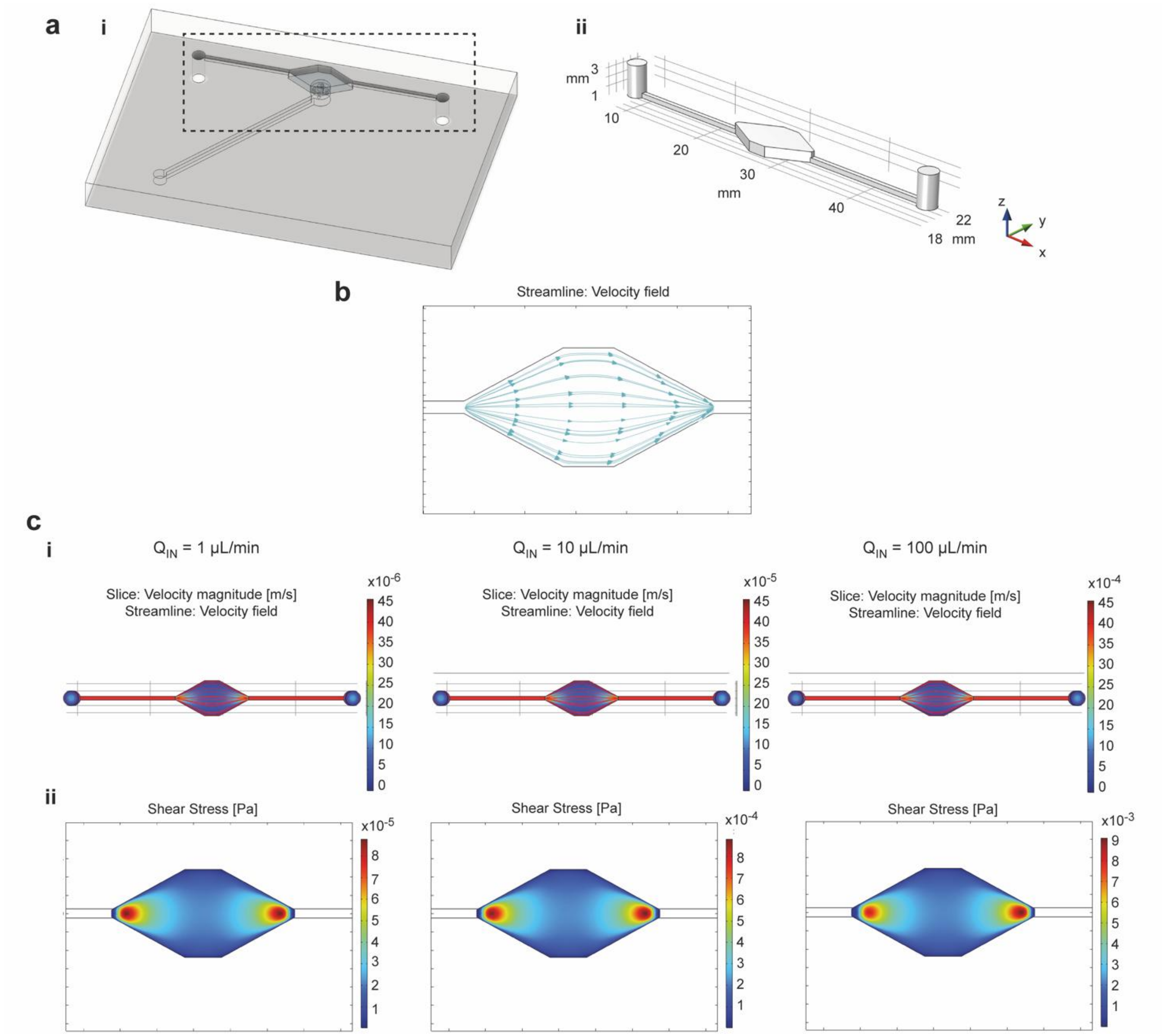

Figure S1. Fluid-dynamic assembly of the stimulation platform. (a) Schematic of the microfluidic device structure (i), with an inset of the channel for the biochemical cues, cell seeding and nutrients supply flow (ii). (b) COMSOL simulation of the fluid streamlines in the cell culture chamber of the microfluidic platform. The geometric shape of the chamber favors laminar flow streams, avoiding the formation of turbulences and shear stresses harmful for cell survival. (c) Fluid flow dynamics in the biochemical stimulation channel was simulated by using COMSOL simulations of the fluid velocity (i) and shear stresses (ii) at different inlet flow rates. The shape and aspect ratio of the chamber and channels allows to obtain laminar flow conditions and low shear stresses at the center of the culture chamber, meaning that no shear forces contribute to the cell stimulation.

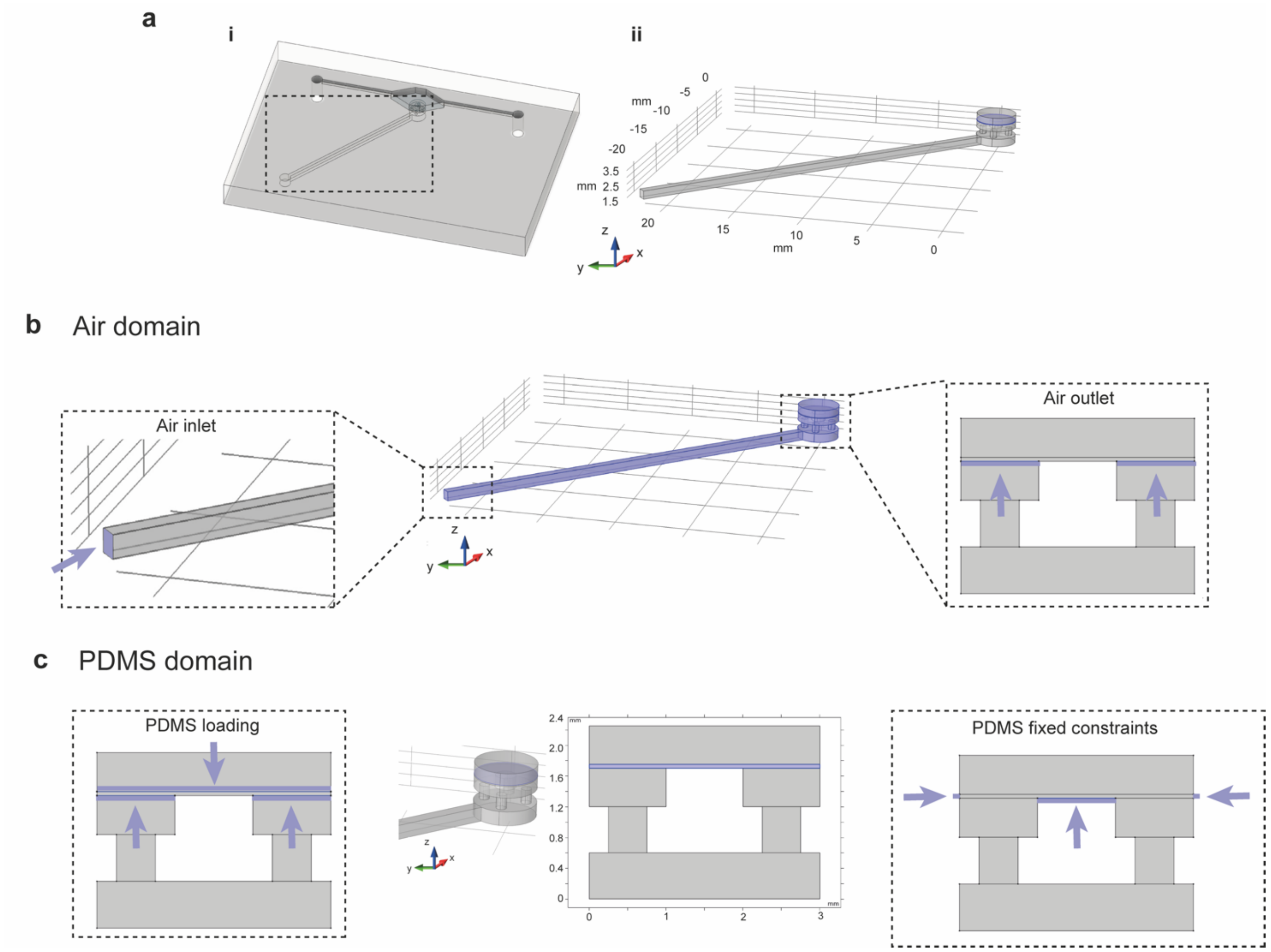

Figure S2. Boundary conditions schematics on the air domain and the PDMS domain, referred to the mechanical stimulation unit COMSOL simulations (a). The Laminar flow interface was used to model the air domain (b), while the Solid Mechanics interface was used to simulate the PDMS domain (c).

Fluid Dynamics (spf): Air (Materials)<br>Laminar flow			
**Fluid Properties**		**From material (37°C)**	
Dynamic viscosity (37°C)	μ_{air}	$1.896 \cdot 10^{-5}$	Pa·s
Density (37°C)	ρ_{air}	1.139	kg/m^3
**Physical model**		**Compressibility: Compressible flow (Ma<0.3)**	
**Wall**		**Boundary condition: No slip**	
**Inlet**	Pressure	P1 = -10	mbar
		**Pressure condition 1: Suppress backflow**	
		**Pressure condition 2: Normal flow**	
**Outlet**	Pressure	0	Pa
		**Pressure conditions 1: Suppress backflow**	
		**Pressure condition 2: Normal flow**	

Table S2. Simulation parameters for the fluid dynamic operation on the air suction in the deformation chamber. The outlet of the air fluid flow was the contact surface between the air camera and the bottom of the PDMS membrane.

Structural Mechanics (solid): PDMS (Materials)<br>Solid Mechanics			
**Material: PDMS**		**From library**	
Density	ρ	970	kg/m^3
Young's modulus	E	200	kPa
Poisson's ratio	ν	0.49	N.D.
**Hyperelastic Material**		**Material model: Neo-Hookean**	
		**Compressibility: Nearly incompressible**	
		**Volumetric strain energy: Quadratic**	
Lamé parameter	μ	1e5	N/m^2
Bulk modulus	κ	5e8	Pa
Density	ρ	970	kg/m^3
**Load on the boundary**		Pressure	From spf
Fixed constraints		Pillar surface	
		PDMS membrane sides	

Table S3. Simulation parameters for the structural mechanics module operation on the PDMS membrane deformation.

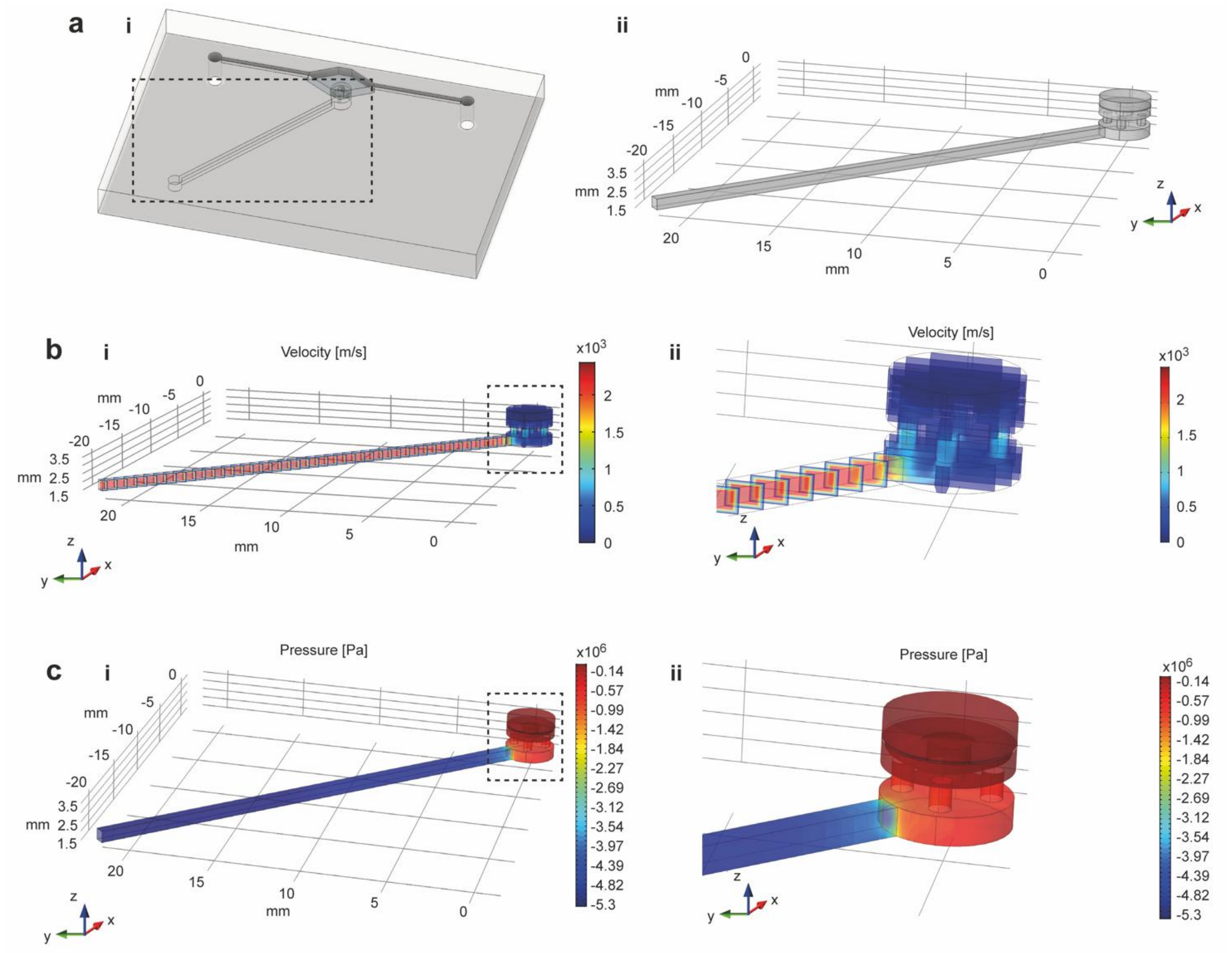

Figure S3. Mechanical stimulation assembly of the microfluidic platform. a) Schematic of the microfluidic device structure (i), with an inset of the channel for the mechanical stimulation, made by a channel for air flow suction and a chamber for the mechanical deformation of the flexible PDMS membrane (ii). COMSOL simulation of the air flow response in the platform helped to quantify air velocity (b) and pressure (c) by applying a negative pressure at the inlet of the air flow channel.

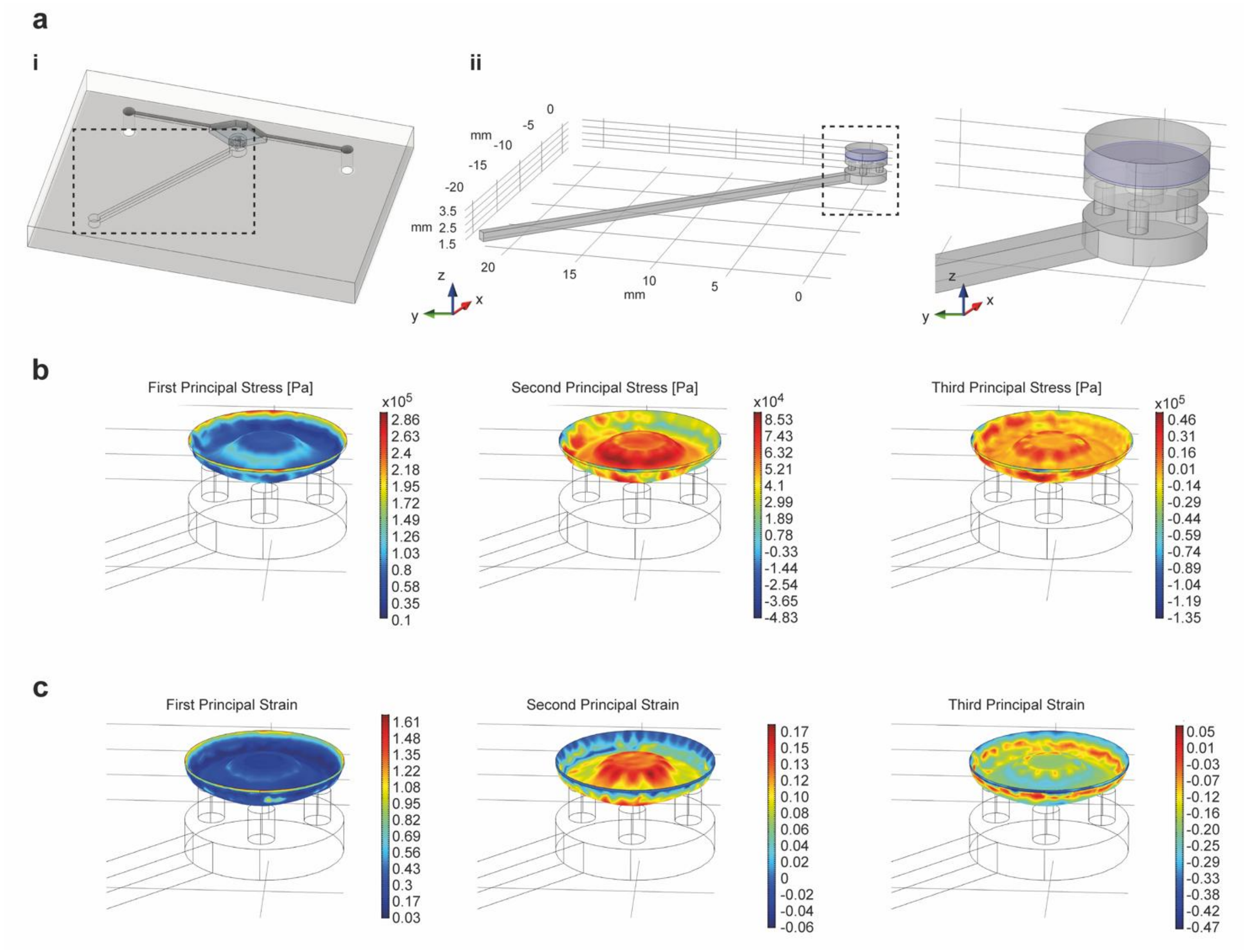

Figure S4. Mechanical response of the PDMS flexible membrane under the action of the air suction. a) COMSOL simulations were applied to the solid mechanics of the PDMS membrane under the deformation effect of the air suction (i), focusing the quantification on the mechanical stimulation unit chamber (ii). The deformation of the membrane was characterized by plotting the principal stresses (b) and strains (c), which represent a measurement of the mechanical status of the deformed body.

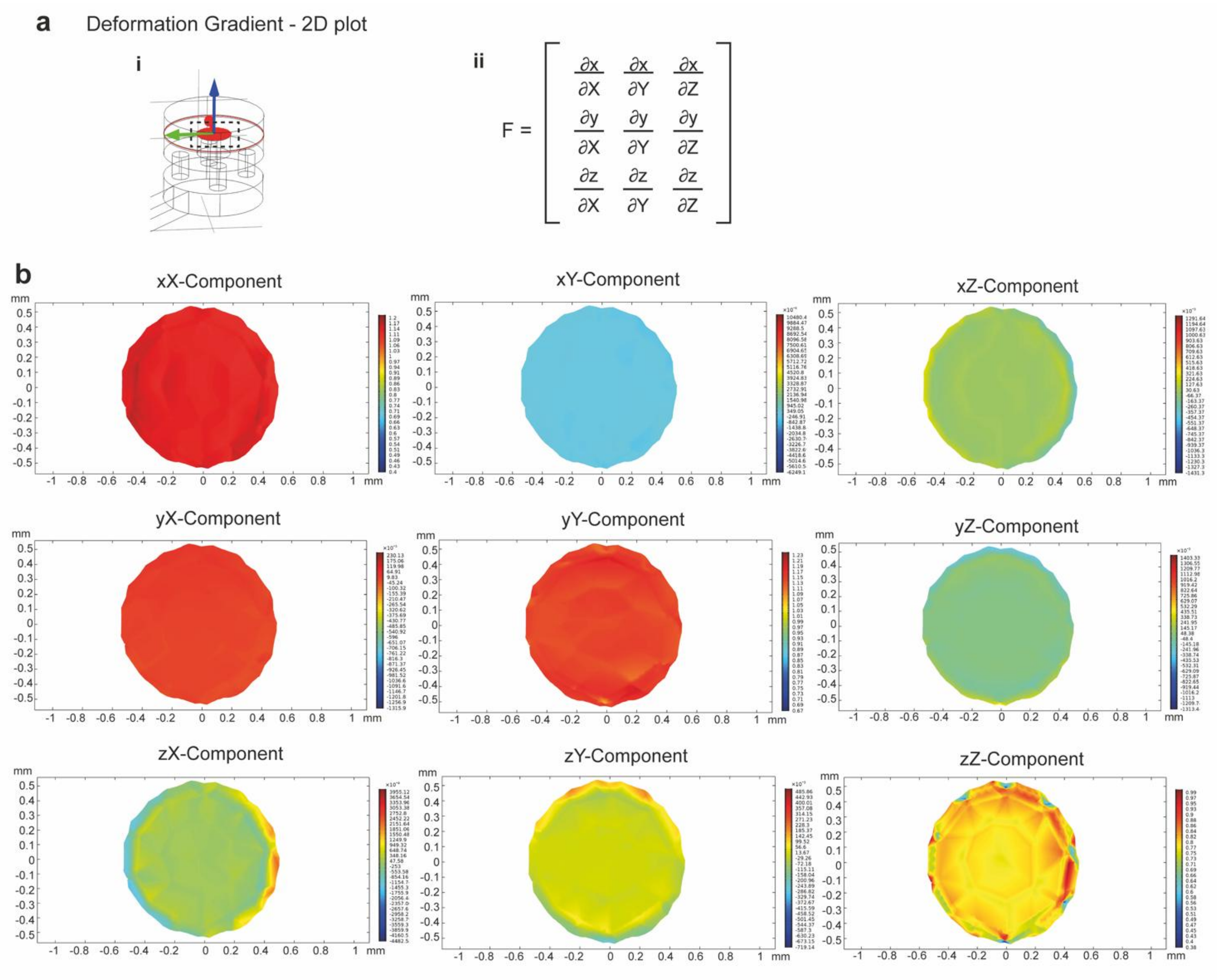

$$F = \begin{bmatrix} \frac{\partial x}{\partial X} & \frac{\partial x}{\partial Y} & \frac{\partial x}{\partial Z} \\ \frac{\partial y}{\partial X} & \frac{\partial y}{\partial Y} & \frac{\partial y}{\partial Z} \\ \frac{\partial z}{\partial X} & \frac{\partial z}{\partial Y} & \frac{\partial z}{\partial Z} \end{bmatrix}$$

Figure S5. The deformation gradient of the PDMS membrane under stretching gave a measurement of the transformation arising in the structure. (a) The deformation gradient was retrieved from simulations above the surface of the PDMS membrane, in correspondence to the circular pillar (i). The matrix definition of deformation gradient tensor F is reported in (ii). (b) 2D plots of the x, y and z components of the deformation gradient highlighted that it was uniform above the pillar surface and the only non-zero components were those on the principal diagonal of the tensor F.

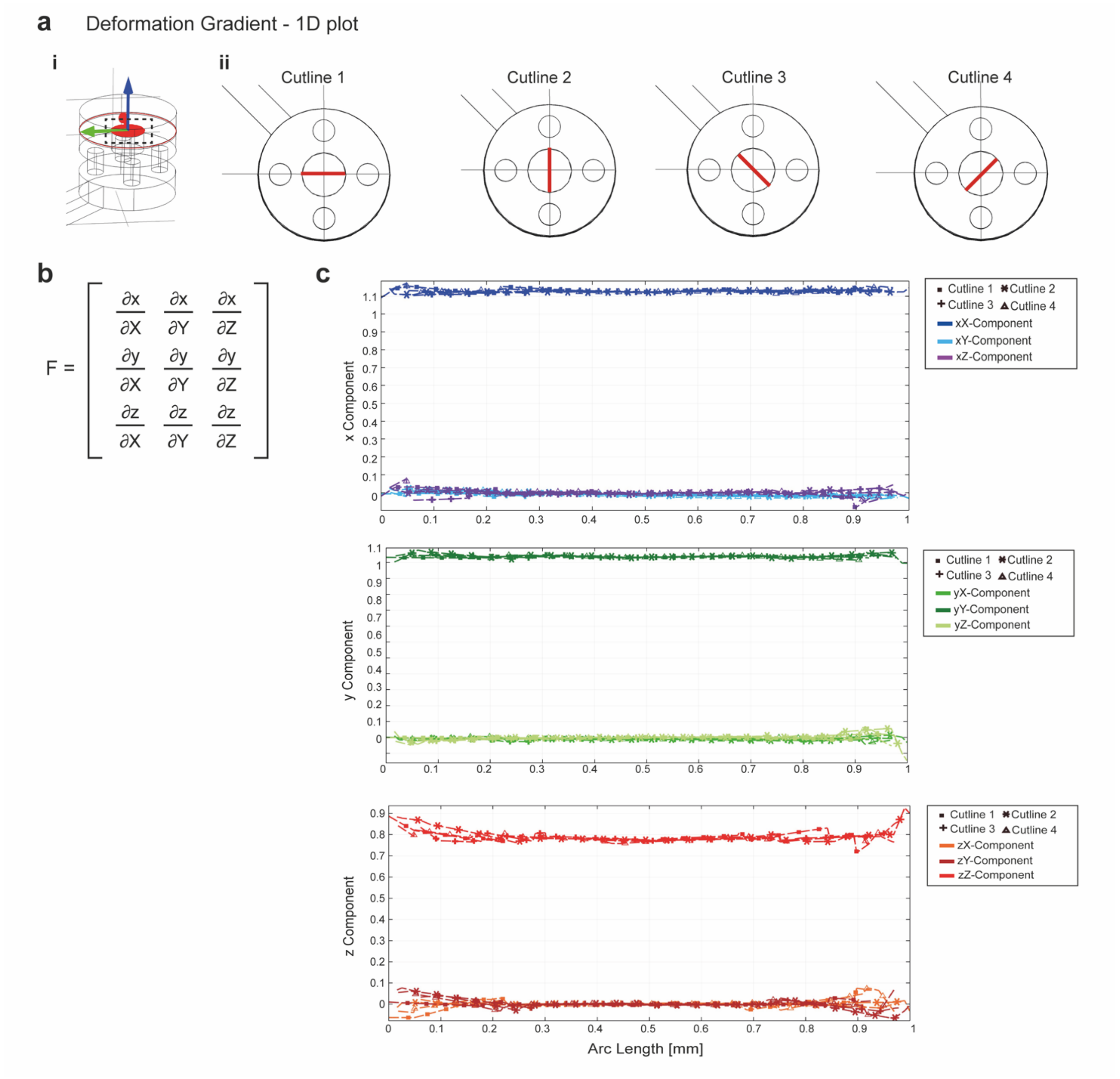

Figure S6. The deformation gradient was also plotted in 1D to quantify how the tensor components distributed above the pillar surface (a-i). Four cutlines were identified in the four principal orientation on the circular pillar, in order to compute the deformation gradient tensor in 1D plots and to identify if a symmetry arose due to the circularity of the pillar (ii). (c) The line graphs of the x, y and z components of the deformation gradient tensor F, defined in (b), confirmed that the deformation gradient distributions in Fig. S5 were uniform above the PDMS surface corresponding to the pillar when a stretch was applied. Again, the only non-null components were those on the principal diagonal of the F tensor.

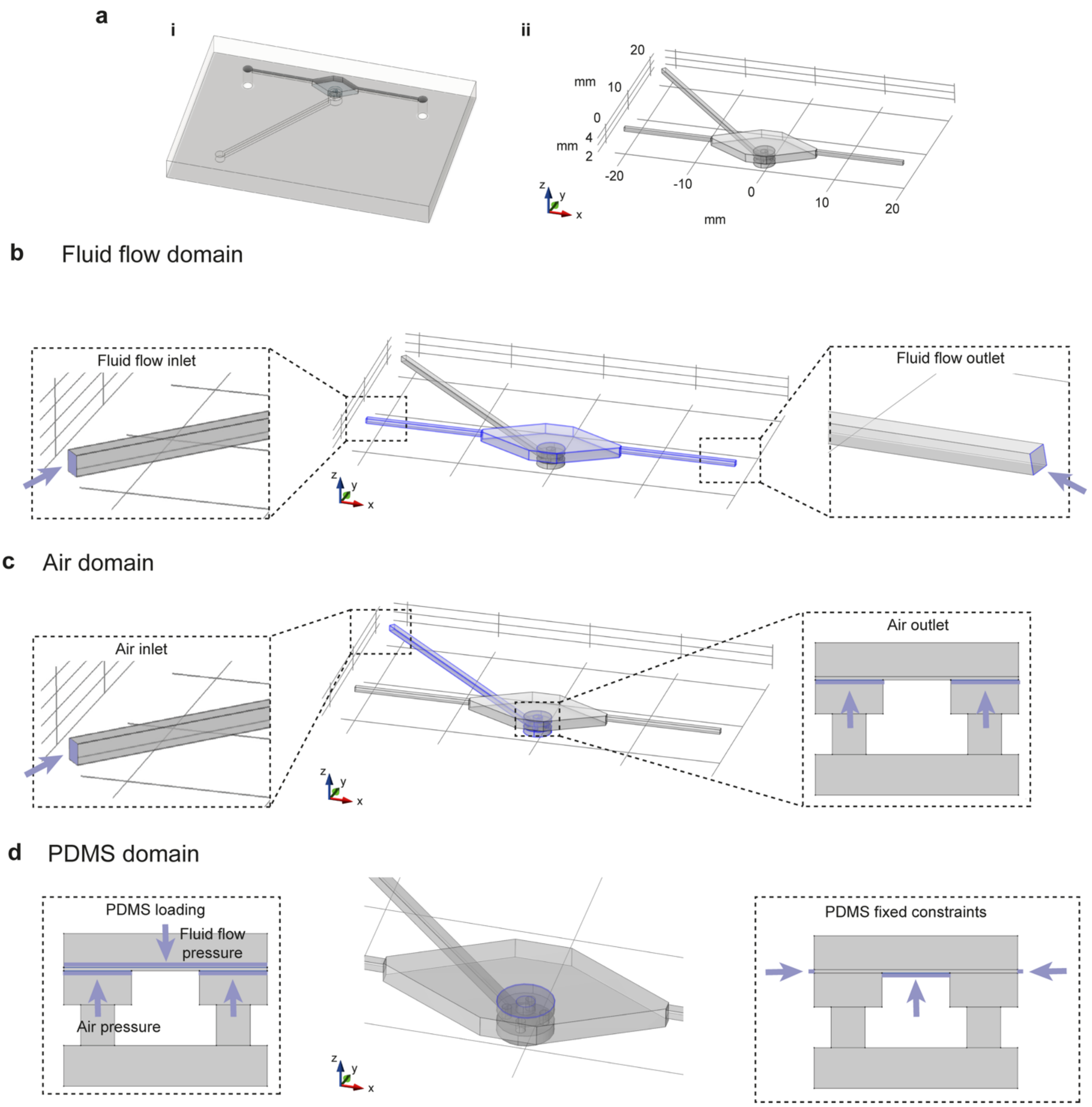

Figure S7. Boundary conditions schematics on the fluid flow domain, air domain and PDMS domain, referred to the mechanical stimulation-fluid flow coupling COMSOL simulations (a). The Laminar flow interface was used to model the air and fluid flow domain (b and c), while the Solid Mechanics interface was used to simulate the PDMS domain (d). Regarding the boundary conditions, the external pressure applied to the PDMS membrane were the air pressure, for the lower boundary, and fluid flow pressure, for the upper boundary.

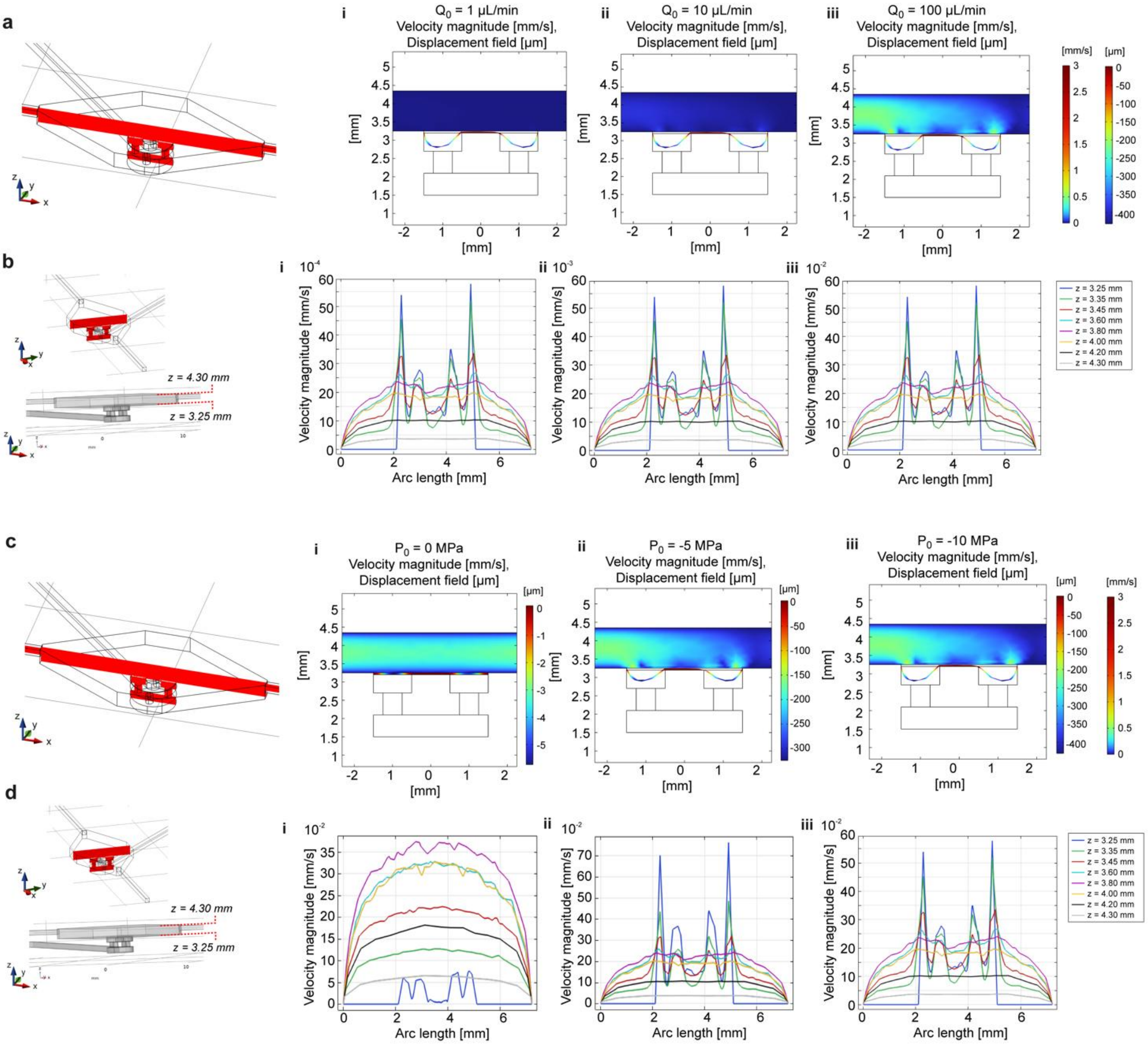

Figure S8. The influence of fluid flow and mechanical stimulation were tested by 3D COMSOL simulations. (a) Inlet flow rate changed (1, 10, 100 µL/min) by fixing the inlet air suction pressure (-10 MPa) and the velocity profile was plotted for a 2D section of fluid flow and mechanical stimulation chamber along with the corresponding membrane deformation. For all the three flow rates (a-i,ii,iii), although the velocity profile changed, the membrane deflection was not affected. (b) The velocity profile was plotted along the thickness of the fluid flow chamber (z = 3.25 mm to z = 4.30 mm) as the inlet conditions changed, demonstrating that the sections far from the membrane in deformation showed the typical parabolic profile, while near the deformed membrane the velocity increased. The values of velocity changed proportionally to the inlet flow rate. (c) The simulations were repeated by keeping constant the inlet flow rate (100 µL/min) and by changing the suction pressure (0, -5, -10 MPa). Since the velocity profile is affected by the deformation, also the 1D profile of fluid flow velocity changes by approaching to the membrane-fluid interface. Instead, the velocity parabolic profile far from the membrane is not affected by the deformation.

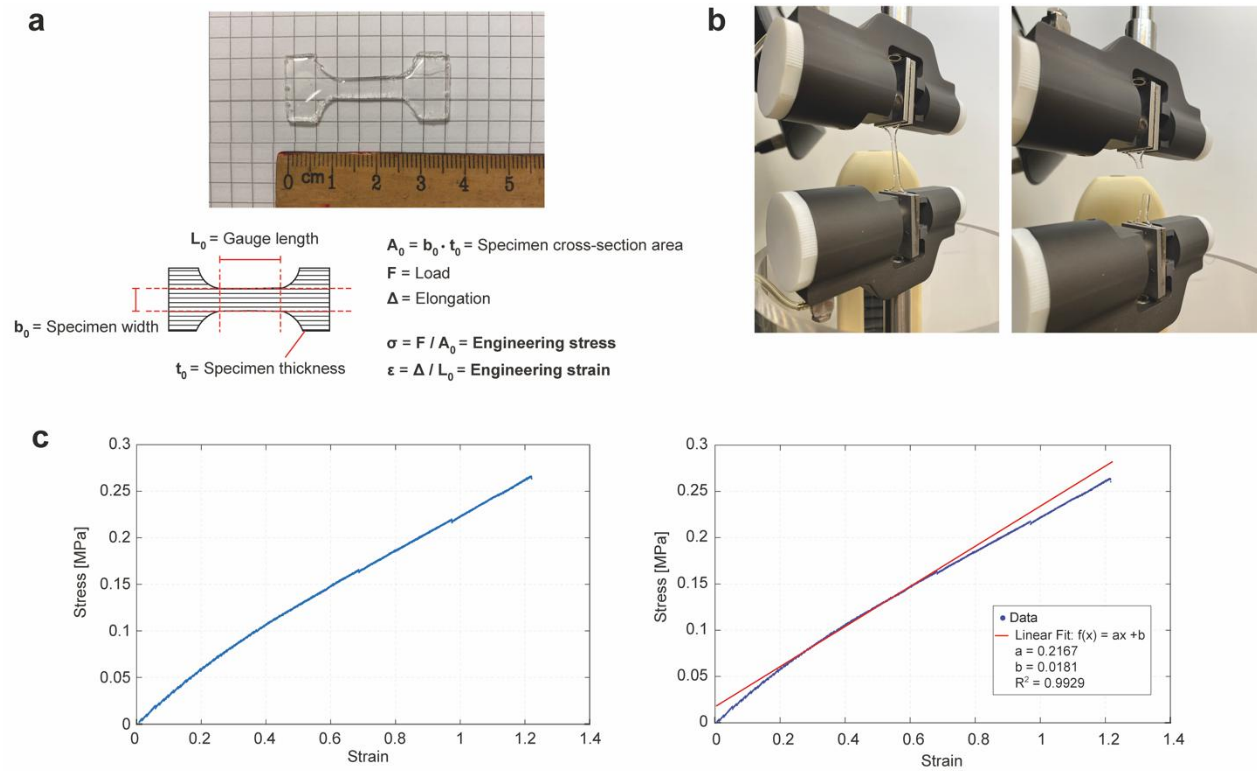

Figure S9. Characterization of the PDMS membrane. Top figures (a) and (b) are the mechanical characterization results of a traction test over a PDMS made in a ratio 20:1. Dog-bone PDMS samples were prepared (a) and they underwent uni- axial traction until rupture (b). Engineering stress-strain curve is fitted in the linear region to retrieve the elastic modulus of the PDMS, which is corresponding to the slope of the linear curve (c). A value of 0.2167 MPa was obtained by the fitting.

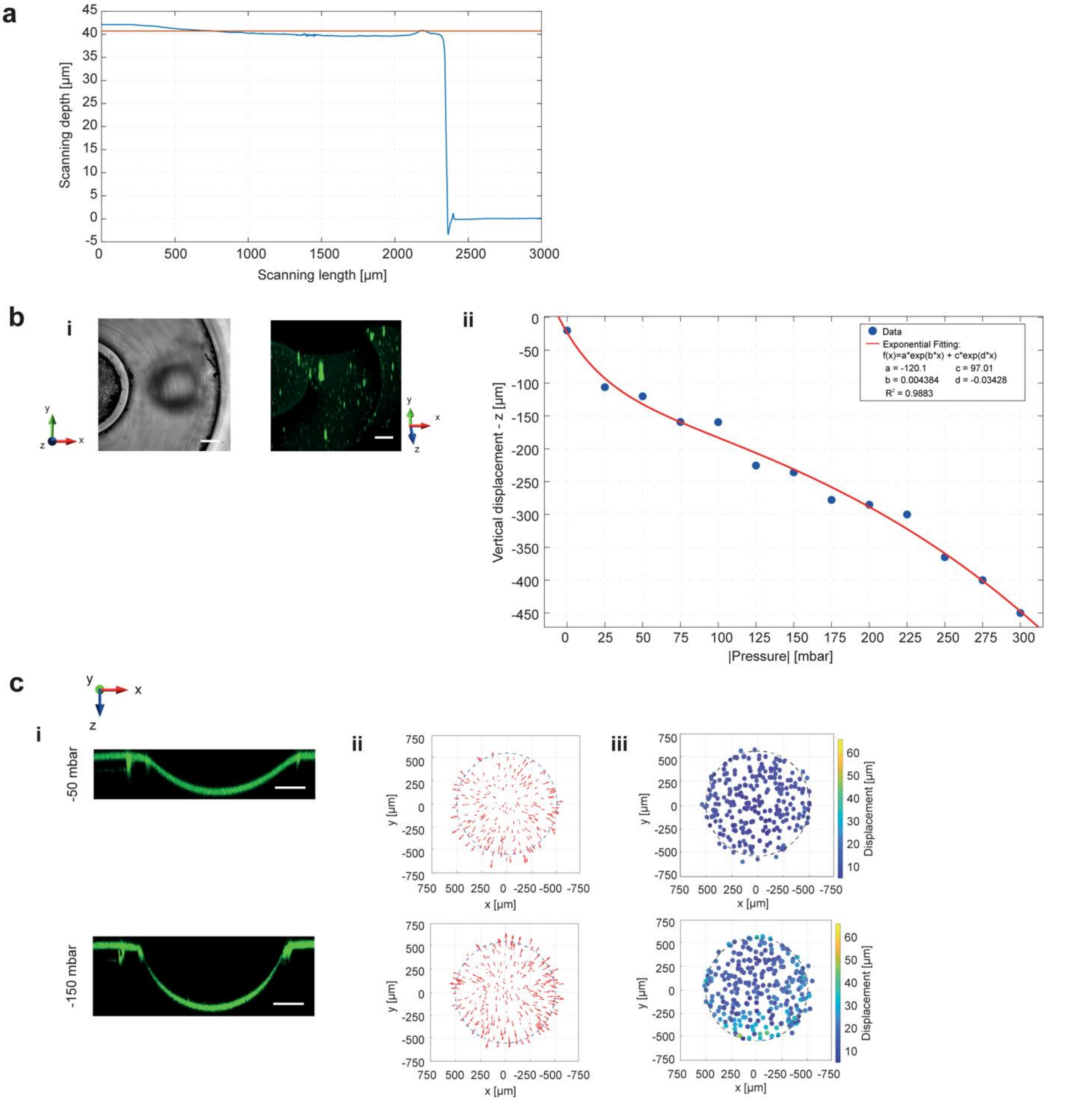

Figure S10. Experimental characterization of the PDMS membrane response to the deformation. (a) A prolifometer characterization of the PDMS membrane thickness was performed. (b) The vertical lowering of the PDMS membrane under deformation was quantified in a calibration curve of the displacement in the suction pressure. The values can be well fitted by an exponential fitting. The parameters of the fitting are reported in the plot. (c) The PDMS membrane sliding over the pillar during equi-biaxial stimulation for intermediated values of air suction pressure (-50 and -150 mbar) are reported. The displacement vectorial field (ii) and the deformation map (iii) changed correspondingly to the negative pressure applied to the membrane.

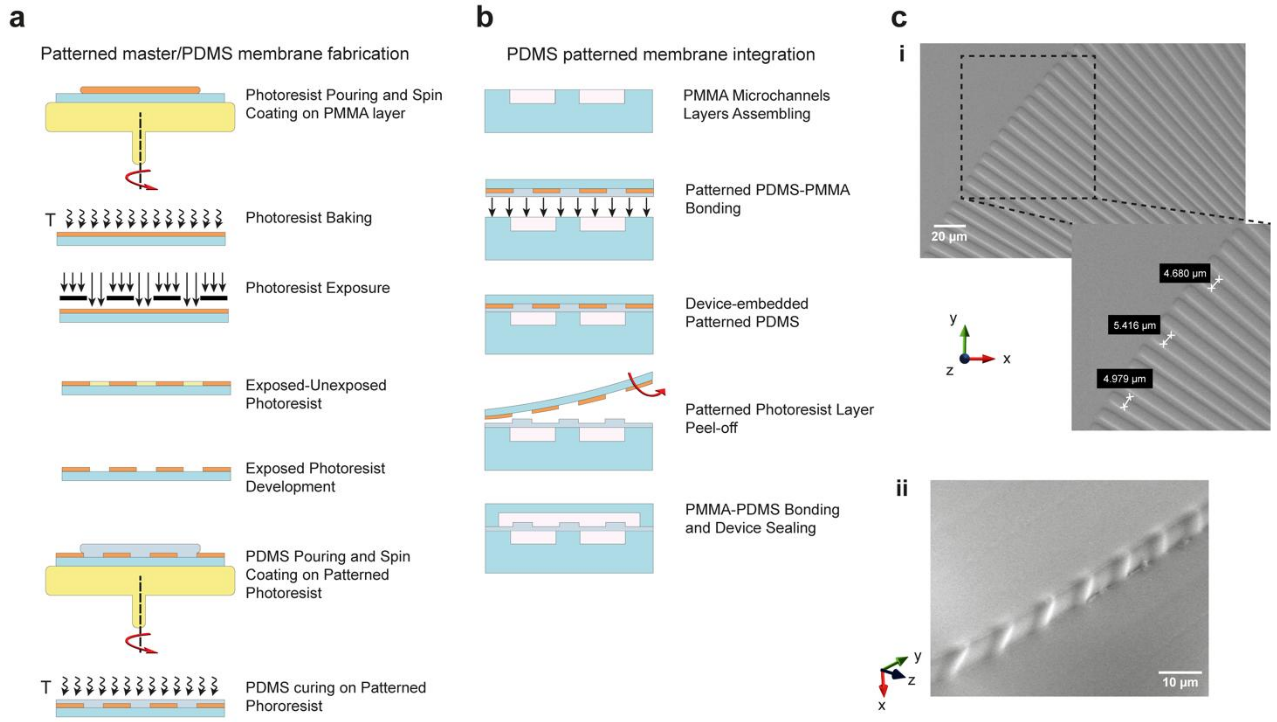

Figure S11. Fabrication of the patterned master and PDMS mold. (a) Schematic of the workflow for the patterned master photoresist on flexible PMMA substrate and PDMS replica molding to obtain the patterned PDMS deformable membrane. (b) Workflow of the integration of the patterned PDMS membrane in the microfluidic device. (c) SEM images of micro- patterned PDMS, with an inset on the dimension of the ridges (i), and of a cryo-sectioned slice of the patterned PDMS, showing the lateral section of the ridges (ii).

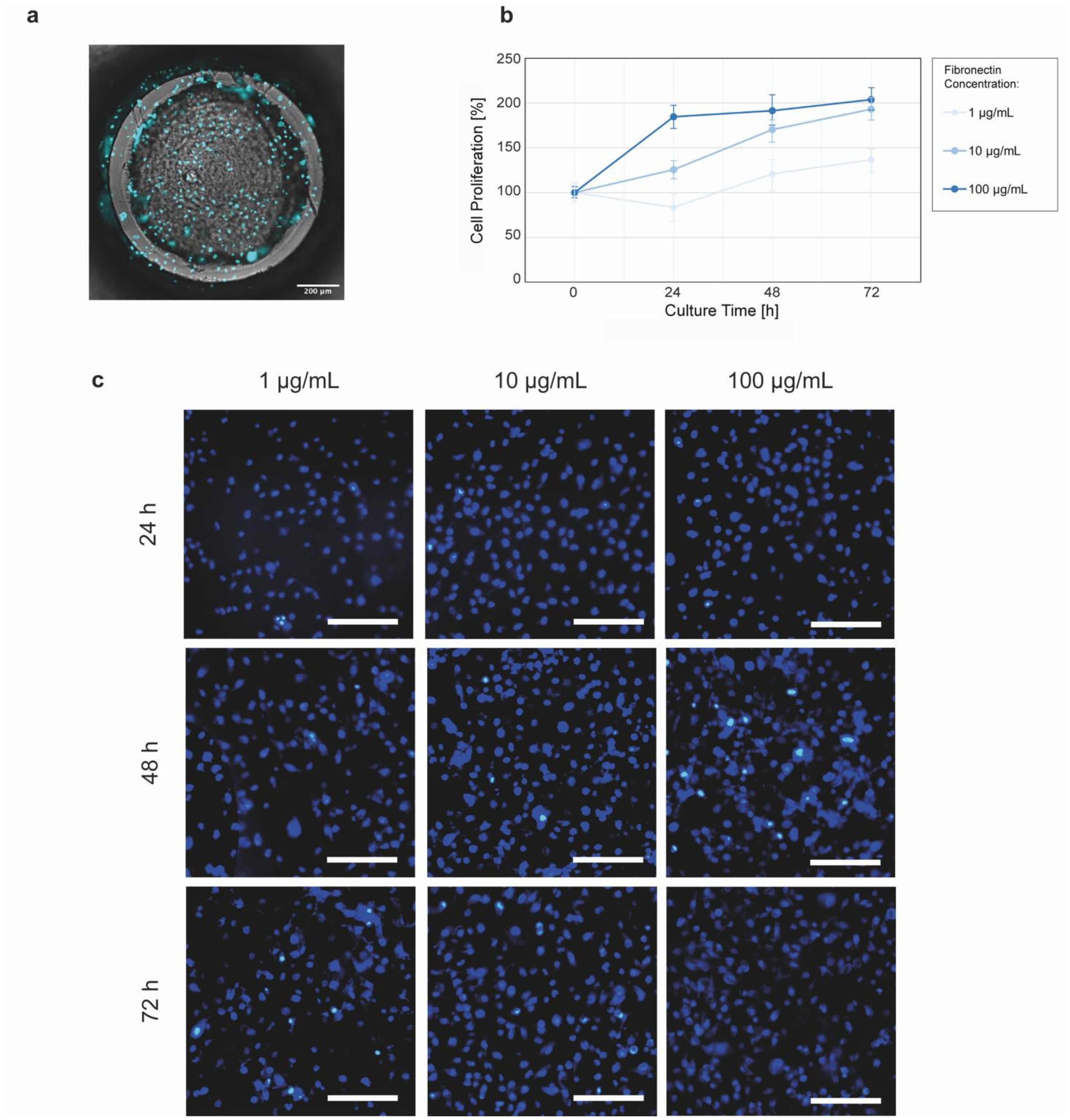

Figure S12. Fibronectin PDMS functionalization improved HL-1 cell proliferation. (a) HL-1 cells nuclei were stained with Hoechst 33342 (cyan) and tracked on the pillar surface when they were seeded in the microfluidic platform. (b) Nuclei count was evaluated from 24 h to 72 h of HL-1 after PDMS functionalization with different concentrations of fibronectin, to improve cell adhesion. By varying fibronectin concentration, the cells proliferation rate changed. Values of proliferation related to 100% corresponded to the cells seeding density at the starting point (time 0). EVOS images of time evolution from 24 h to 72 h of HL-1 nuclei stained with Hoechst 33342 (blue) and seeded on PDMS functionalized with different concentrations of fibronectin. Scale bar equal to 200 µm.

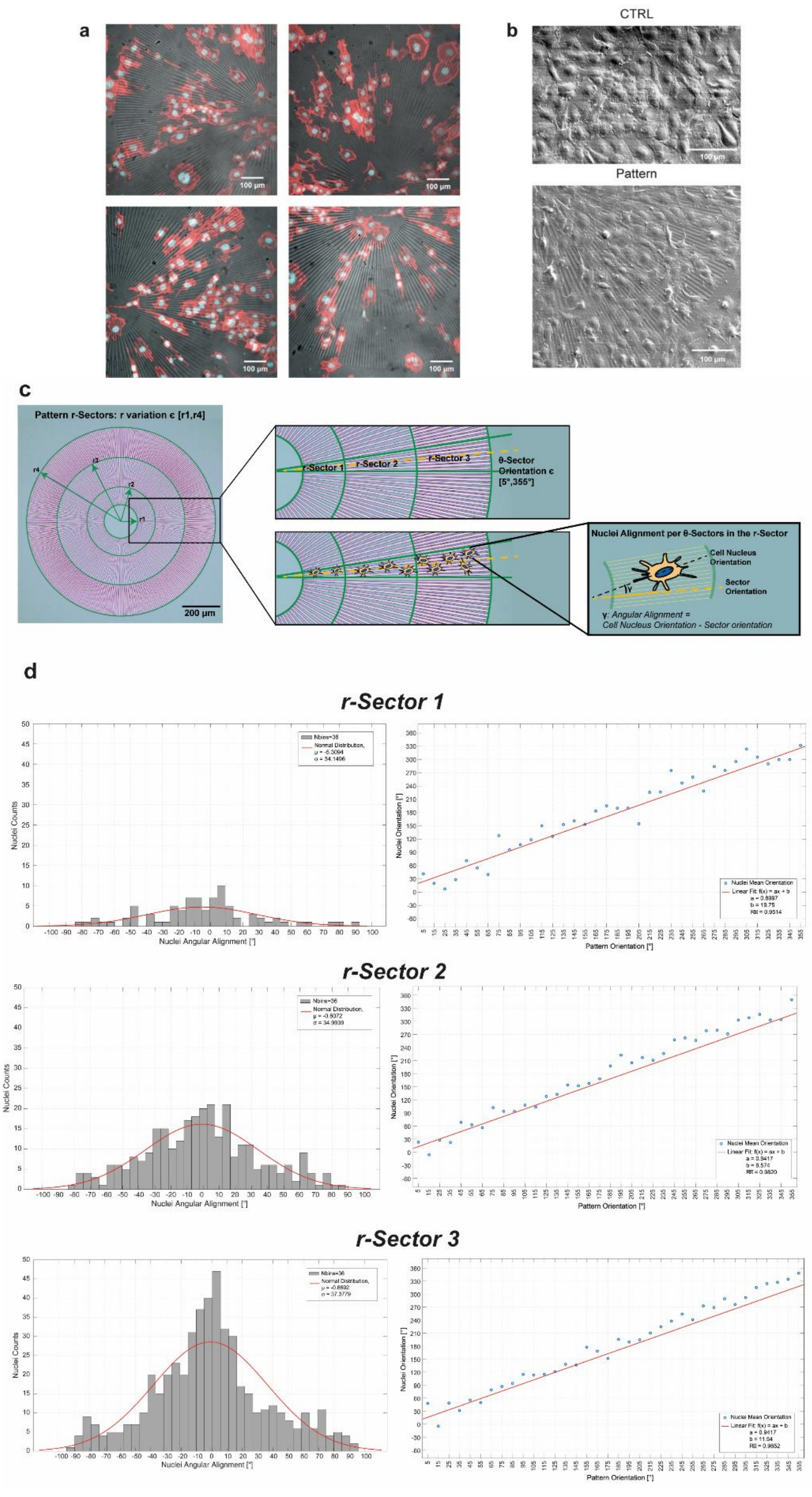

Figure S13. The radial micro-pattern guides HL-1 cells alignment. a) Confocal images of HL-1 cells seeded for 24 h on patterned PDMS show that cells nuclei and f-actin aligned towards the patterned stripes in every section of the circular topography area. b) SEM images of cells seeded on flat PDMS (CTRL) and radially patterned PDMS further confirm that HL-1 oriented along the pattern direction, differently from the random arrangement followed when they were seeded over flat samples. c) Logic of nuclei alignment data analysis when deformation gradient was applied to cells seeded on the micropattern. The radial micro-pattern was divided into sectors of 10° and, in each of them, sectors along the r-direction were recognized from the deformation gradient map in Fig. 2. Here nuclei orientation was compared to the mean orientation of the belonging sector, retrieving in this way the angular alignment for the cell. d) Distributions of cells angular alignment in the r-sectors and fitting curves of cells orientation versus pattern orientation in each θ-sector for the three r-sectors were derived for nuclei demonstrating the ability of the pattern to align HL-1 CMs, despite the deformation gradient arising in the radial direction.

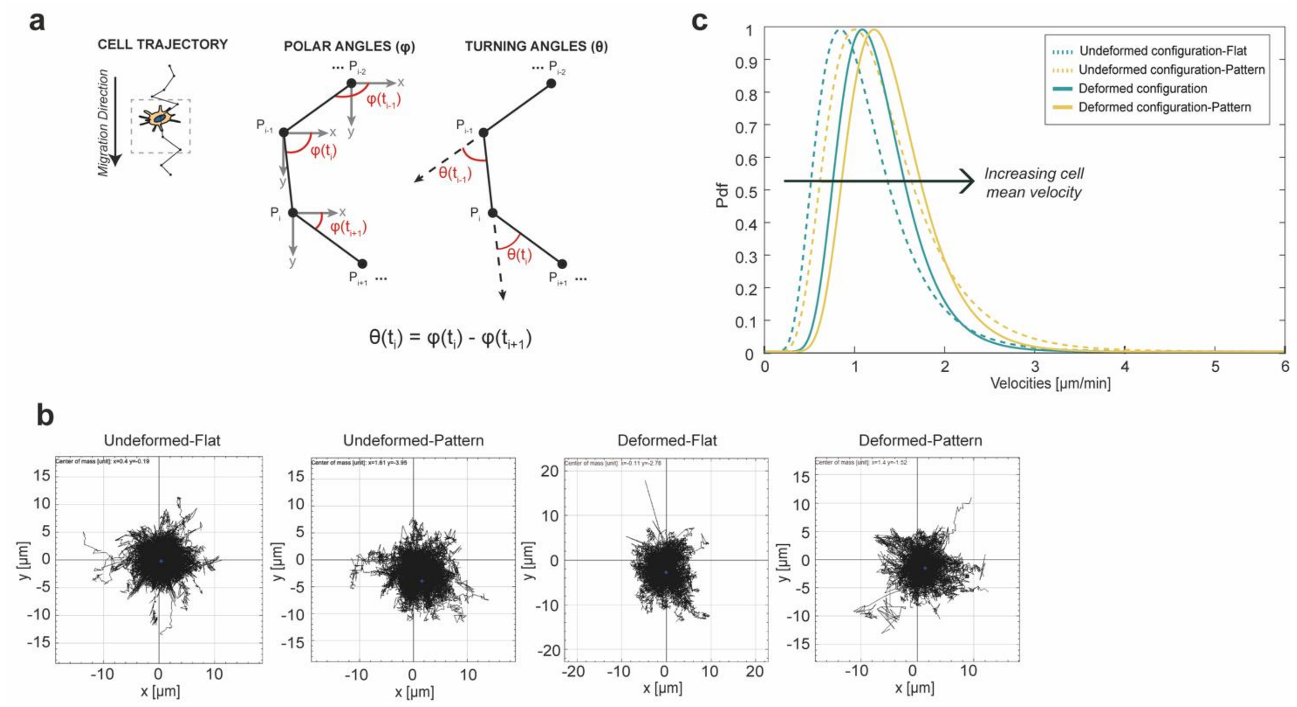

Figure S14. HL-1 cells migration response under mechanical deformation on flat and patterned substrate. a) Cell trajectories tracking was used to compute the migration direction of the cardiac cell line. Turning angles were computed as the difference of consecutive polar angles, indicating the ability of the cell to maintain a straight path during the migration. b) Cell trajectories arrangement on the x-y plane for HL-1 cells migrating on undeformed and deformed flat and patterned substrate. c) Distribution of migration velocity for cells migrating on flat and patterned substrate both with and without mechanical deformation.

group_A	group_B	V	p_raw	p_holm	reject_at_0.05
Undeformed Flat	Deformed Flat	0.283	0.0	0.0	TRUE
Undeformed Flat	Deformed Pattern	0.322	0.0	0.0	TRUE
Undeformed Pattern	Deformed Flat	0.283	0.0	0.0	TRUE
Undeformed Pattern	Deformed Pattern	0.328	0.0	0.0	TRUE
Deformed Flat	Deformed Pattern	0.0845	3.55e-40	7.10e-40	TRUE
Undeformed Flat	Undeformed Pattern	0.0372	1.56e-11	1.56e-11	TRUE

Table S4. Statistical analysis results on comparisons between turning angles distributions. V is the statistics, p raw is the significance, p holm is the corrected significance.

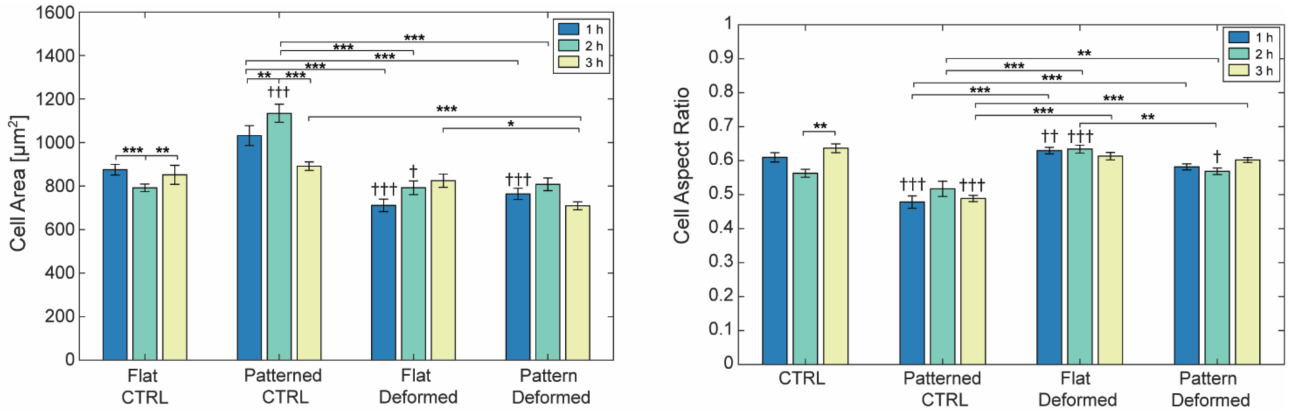

Figure S15. HL-1 cells morphological adaptation to biophysical cues. In the case of flat substrates, cells experienced a slight reduction in cell area in the first hour of stretching, with a following increase after 3 h. In patterned substrates in non-deformed configuration, cells showed larger area due to increased contact guidance during the interaction with the micropattern, while the application of mechanical deformation is affecting significantly the cells' spreading reducing their area (less stable focal adhesions mean less pulling and spreading of cells). Regarding the cell body aspect ratio, before deformation, the cells are polarized over patterned substrates, while the mechanical stimulation influences the polarity of cells seeded on patterned substrate. A slight reduction of polarity can be observed, maybe associated to the adaptation phenomena that cells are experiencing in the competing effect of topography and stretching (cell increased movement towards the strain gradient means that cells are continuously disassembling and reassembling focal adhesions, resulting in a disruption of polarity for cell body reorientation).

P values are:

* p<0.05

** p<0.01

*** p<0.001

† statistical difference of the column with the corresponding time point of Flat CTRL

Aspect ratio was defined as follows:

$$Aspect\ ratio = \frac{Cell\ Minor\ Axis}{Cell\ Major\ Axis}$$
